# On the formation of anions: frequency-, angle-, and time-resolved photoelectron imaging of the menadione radical anion†
†Electronic supplementary information (ESI) available: A summary of the ground-state geometries and molecular orbitals from the *ab initio* calculations; fitted residuals from the FA-PI simulation; plots of all spectra included in the frequency-resolved two-dimensional figure; and example time-resolved PE spectra from the 3.10 + 1.55 eV pump-probe experiments. See DOI: 10.1039/c4sc03491k



**DOI:** 10.1039/c4sc03491k

**Published:** 2014-12-17

**Authors:** James N. Bull, Christopher W. West, Jan R. R. Verlet

**Affiliations:** a Department of Chemistry , Durham University , South Road , DH1 3LE , UK . Email: j.r.r.verlet@durham.ac.uk

## Abstract

Frequency-, angle-, and time-resolved photoelectron imaging of gas-phase menadione (vitamin K_3_) radical anions was used to show that quasi-bound resonances of the anion can act as efficient doorway states to produce metastable ground electronic state anions on a sub-picosecond timescale. Several anion resonances have been experimentally observed and identified with the assistance of *ab initio* calculations, and ground state anion recovery was observed across the first 3 eV above threshold. Time-resolved measurements revealed the mechanism of electronic ground state anion formation, which first involves a cascade of very fast internal conversion processes to a bound electronic state that, in turn, decays by slower internal conversion to the ground state. Autodetachment processes from populated resonances are inefficient compared with electronic relaxation through internal conversion. The mechanistic understanding gained provides insight into the formation of radical anions in biological and astrochemical systems.

## Abstract 2

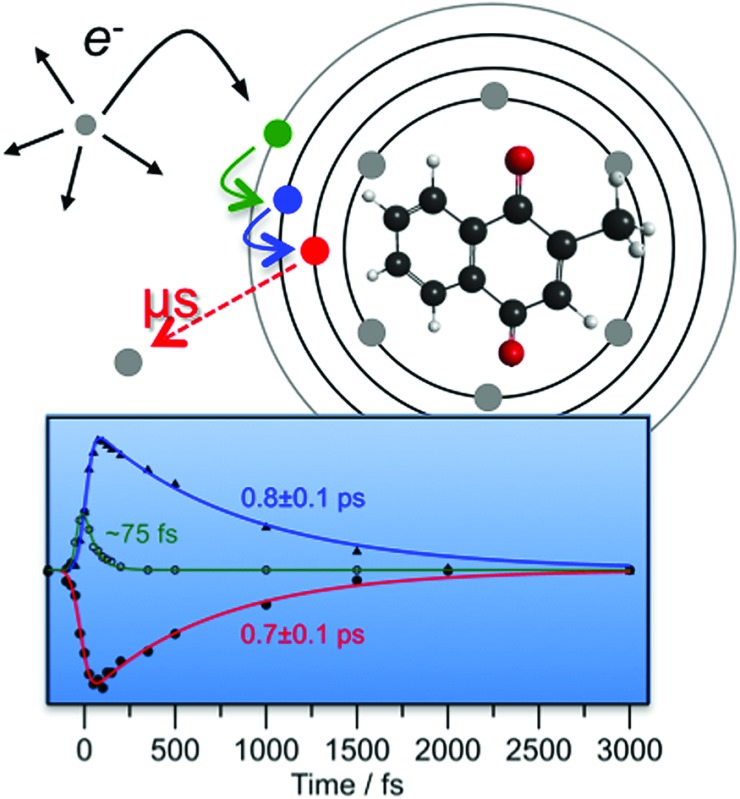
Frequency-, angle-, and time-resolved photoelectron imaging of gas-phase menadione (vitamin K_3_) radical anions is used to show that quasi-bound resonances of the anion can act as efficient doorway states to produce metastable ground electronic state anions on a sub-picosecond timescale.

## Introduction

Electron attachment is one of the most fundamental classes of chemical reaction, and a wide variety of important scientific and technological processes rely on a detailed understanding of this process.^1^ Examples include reactions in the atmosphere and ionosphere,^2^ astrochemical processes such as anion formation in the interstellar medium,^3^ semiconductor etching,^4^ and electron transfer reactions.^5^ Despite the importance of understanding electron attachment mechanisms, probing the primary steps or intramolecular dynamics immediately following acceptance of an electron into specific orbitals of a neutral molecule is a challenging task. In this paper we demonstrate that frequency-, angle-, and time-resolved photoelectron imaging (FAT-PI) of a radical anion can provide deep insight into the elementary steps of anion formation, and we apply this methodology to understand the electron resonances of isolated menadione molecules.

**Fig. 1:**
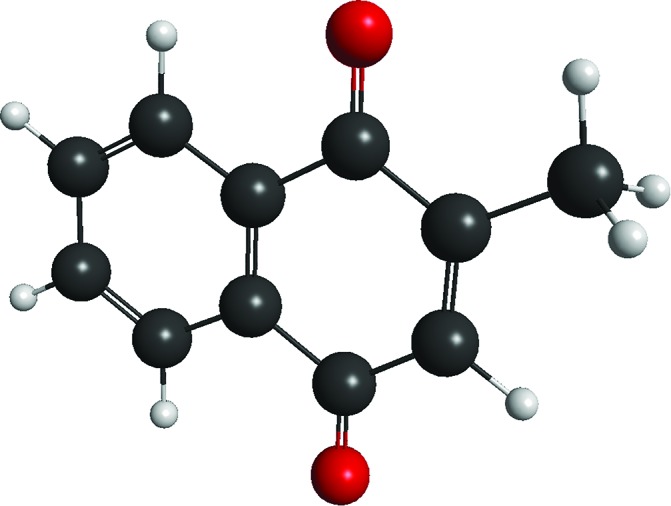
Illustration of the menadione molecule with *C*_S_ point symmetry. Key: charcoal – carbon; red – oxygen; and white – hydrogen.

Menadione (2-methyl-1,4-naphthoquinone), also commonly known as vitamin K_3_ (Fig. 1), is the precursor and the key structural subunit in the naturally-occurring vitamins K_1_ and K_2_.^6–10^ Vitamin K_1_ is synthesised by plants and plays an important role as an electron acceptor in the electron transport chain of the botanic photosystem. Vitamin K_2_ plays an electron transport role in mammalian cells. In general, an understanding of the anionic electronic structure and electron attachment/detachment dynamics of small structural moieties, which represent the active centres in larger biological species, can provide a route to understanding more complex biological systems. Molecular systems such as menadione also provide valuable information in understanding how anions are formed. For example, strong infrared emissions originating from the interstellar medium can be traced to the vibrational relaxation of UV-excited polycyclic hydrocarbons,^11–14^ amongst which anionic polycyclic hydrocarbons and carbon rich systems have also been identified. Such anions are presumably formed through capture of free electrons. The key question is: how does electron-capture lead to a stable anion?

The radical anion of menadione is arguably structurally and electronically similar to some polycarbon radical anions, and hence represents a prototypical-carbon rich moiety.

Resonances or temporary anions are quasi-bound states that are energetically situated above the neutral and are thus formally unbound to electron ejection.^15–17^ Such resonances represent excited anion states if the parent molecule exhibits a stable valence-bound anion. Temporary anions are formed through capture of a low energy free electron into a suitable unoccupied orbital; a combination of polarization, electron exchange, and centrifugal barrier effects lead to the electron being trapped for a short duration (tens to hundreds of femtoseconds). Following the initial attachment, several above-threshold processes may be available, including: prompt autodetachment, molecular dissociation, or direct electron ejection following a repulsive potential energy surface; delayed autodetachment or dissociation, either following some nuclear rearrangement on the initially excited state or from some other resonance that can be populated following internal conversion; and the formation of the anionic ground state through internal conversion, which can subsequently lead to slow thermal electron emission or dissociation. The plethora of excited state processes occurring on the inherently short lifetimes of anionic resonances means that the resonances and their interconversion dynamics are difficult to study experimentally. Nevertheless, we have recently demonstrated how time-resolved photoelectron (PE) spectroscopy can be used to monitor the above-threshold dynamics of resonances in *para*-benzoquinone (*p*BQ),^18^ and how frequency- and angle-resolved PE spectroscopy provides signatures of these dynamics.^19^

In this paper, we present a comprehensive account of the resonances and associated dynamics of gas-phase menadione anions using FAT-PI combined with excited state *ab initio* electronic structure calculations. In contrast to conventional electron attachment experiments, the present experimental approach considers photo-excitation of the ground electronic state anion to prepare temporary anions; the ejected electrons measured using PE imaging provides information concerning accessible decay pathways.^19^ A key advantage of optical preparation of the anion resonance is that it enables the dynamics of the resonances to be followed in real-time using time-resolved PE imaging.^20–23^ One of the major results from the present work compared with the smaller and structurally-related *p*BQ species is a greatly enhanced yield of metastable ground state menadione radical anions, which arises from its larger size and orbital delocalization along with the presence of a bound excited state.

## Experimental

All experiments were performed using a PE imaging spectrometer that has been previously described in detail.^24^ Briefly, a ∼1 mM solution of menadione in methanol was electrosprayed and transferred *via* a vacuum transfer capillary into an RF ring-electrode ion trap. A similar solution of vitamin K_1_ dissolved in toluene was also prepared. Electrospray of radical anions is usually more difficult compared with closed-shell (*e.g.*, deprotonated) anions, however no special conditions were required for these quinones. The trap is unloaded into a co-linear time-of-flight optics assembly in which the ion packet is accelerated along a 1.3 m flight region towards a continuous-mode penetrating field velocity-mapping (VMI) assembly.^25,26^ Laser pulses are timed to interact with the mass-selected ion packet at the centre of the VMI stack. Ejected electrons are velocity-mapped onto a dual (chevron) multichannel plate detector followed by a P43 phosphor screen. Electron impacts were monitored by a CCD camera utilizing a ∼500 × 500 pixel array. The PE kinetic energy (eKE) scale was calibrated using the PE spectrum of I^–^, and the velocity-mapping resolution is around 5%. All velocity-map image reconstructions used a polar onion peeling (POP)^27^ algorithm, providing the PE spectrum and electron ejection angular distributions.

In the frequency- and angle-resolved (FA) PI experiments, images were collected between *hv* = 4.66 eV (266 nm) and *hv* = 1.77 eV (700 nm) in 5 nm, 10 nm, or 20 nm increments. The tuneable radiation was generated by a Nd:YAG pumped (Continuum Surelite II-10) optical parametric oscillator (Continuum Horizon I). Each image accumulated sufficient counts to achieve a reasonable and comparable signal-to-noise ratio. Images were accumulated with the detector operating under linear gain conditions, and with sufficiently low laser fluence (unfocussed laser beam) to avoid multiple-photon cycling. Spectrally, multiple-photon cycling manifests as a broadening of the lowest energy thermionic emission peak because recovery of the anion ground electronic state can occur faster than the laser pulse duration (∼6 ns) and vibrationally-hot ground electronic state anions can absorb subsequent photons. The avoidance of multiple-photon cycling was carefully checked at 266 nm and 400 nm through comparison of fluence-dependent spectra with those collected using femtosecond laser pulses, for which only a single photon was absorbed. Finally, all images were accumulated with a 500 ns MCP gate, which is sufficient to detect any electron ejection occurring over this timescale. However, as thermionic emission occurs over a timescale of tens of microseconds in this system,^28,29^ only a fraction of the thermionic emission contribution is actually measured.

For the time-resolved (T) PI experiments, femtosecond laser pulses were derived from a Spectra Physics Ti:sapphire oscillator and regenerative amplifier. The 3.10 eV (400 nm, ∼10 μJ) pump pulses were produced by frequency doubling of the 1.55 eV (800 nm) fundamental in a type I β-barium borate (BBO) crystal. As a probe, femtosecond pulses at 0.95 eV (1300 nm, ∼40 μJ) or 1.55 eV (800 nm, ∼40 μJ) were used. Pump and probe pulses were delayed relative to each other, Δ*t*, using a motorised delay line. Both pulses were combined collinearly using a dichroic mirror, and were loosely focused into the interaction region using a curved metal mirror. Combined, the pump-probe cross-correlation has a full width at half maximum of ∼70 fs. The 3.10 eV pump was operated with a sufficiently low fluence to minimise multiphoton detachment. Much of the multiphoton signal was traced to the fact that two 3.10 eV photons lead to the formation of excited neutral states with an apparently very large cross-section. We note that this contribution should be constant across all Δ*t*, and will therefore be cancelled by appropriate background subtraction.

The T-PI experiments using a 3.10 eV pump and 1.55 eV probe also induce dynamics in the reverse direction – that is to say, 1.55 eV pump and 3.10 eV probe. The convention used throughout this paper is that positive Δ*t* corresponds to 3.10 eV acting as the pump pulse.

## Computational

All *ab initio* calculations were performed using the GAMESS-US (May 2013 release) package.^30^ Ground and excited state calculations were performed within the multi-state second-order XMCQDPT (extended multiconfigurational quasi-degenerate perturbation theory)^31^ framework assuming (17,14) and (16,14) CASSCF reference wavefunctions. That is, 17 or 16 active electrons distributed amongst 14 active orbitals for the neutral and anion, respectively. These active spaces include all π and π* molecular orbitals as well two oxygen lone pair orbitals (see ESI†). Relevant geometry optimisations and harmonic vibrational frequency calculations were performed with the CASSCF wavefunctions. Zero point energies were scaled by factor 0.98.^32^ Oscillator strength calculations assumed the state averaged CAS–CI framework. All calculations used the aug-cc-pVDZ basis set,^33^ although for computational tractability exclude the most diffuse set of *d* functions centred on carbon atoms. Menadione does not support a dipole-bound anion.^34^

Similar excited state calculations were also performed on methyl-*para*-benzoquinone, denoted m-*p*BQ, which can be correlated with earlier calculations on the *p*BQ subunit.^19^

## Results and analysis methodology

### Frequency- and angle-resolved PE spectroscopy

**Fig. 2:**
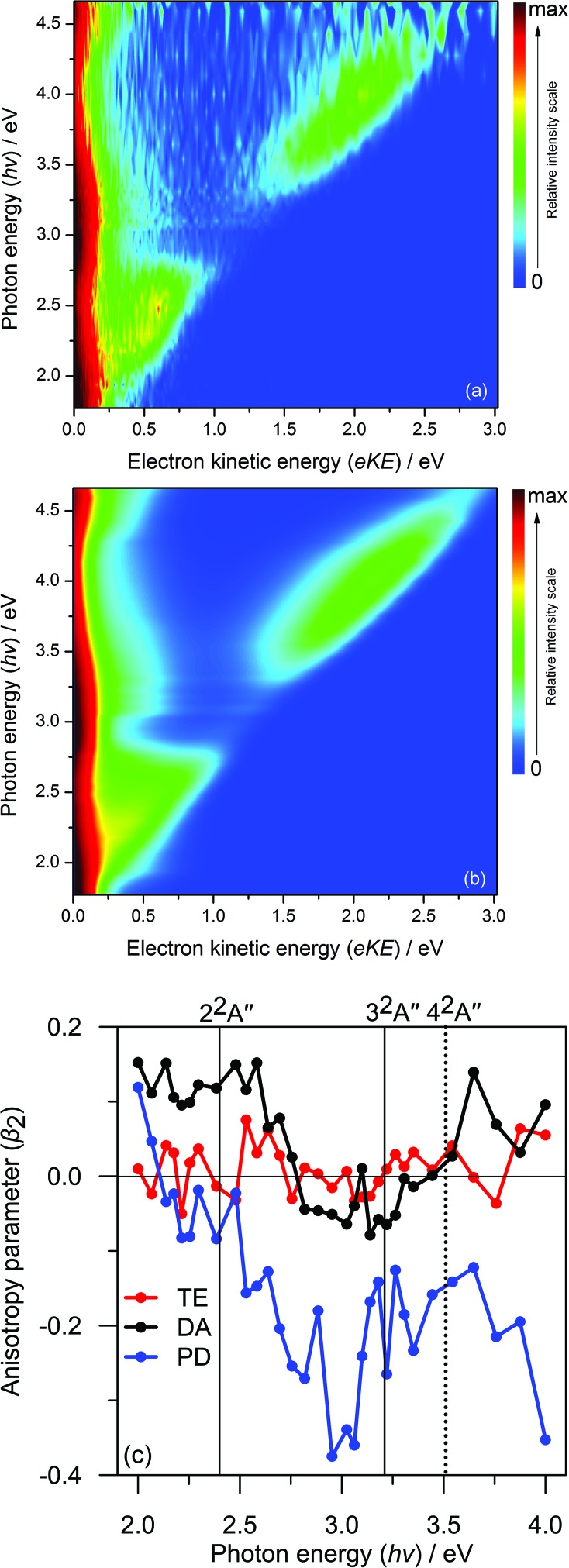
(a) Frequency-resolved photoelectron spectra of menadione; (b) globally-fitted model of experimental data; and (c) *β*_2_ anisotropy parameters averaged over each detachment channel. Further details are given in the text.

In total, the FA-PI spectra consist of 41 individual PE spectra, which are shown in Fig. 2(a) as a false-colour contour plot (individual spectra are available in the ESI†). In Fig. 2(a), each PE spectrum is normalised such that its total area is unity.

**Fig. 3:**
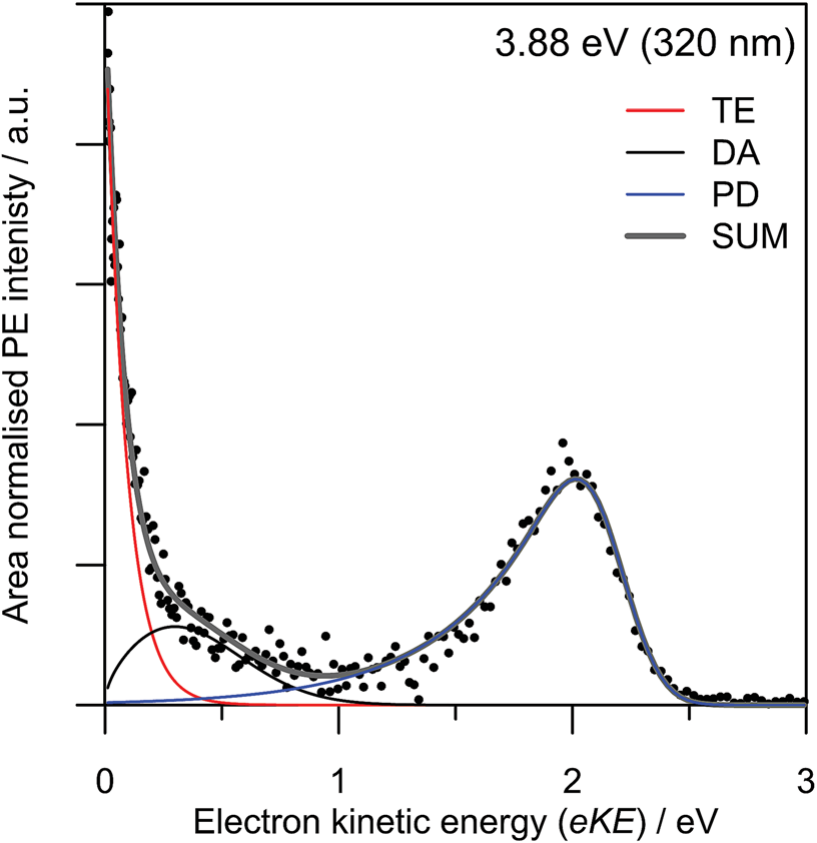
Representative photoelectron spectrum at *hv* = 3.88 eV (320 nm) and model fit, based on three components: TE is thermionic emission; DA is delayed detachment; PD is prompt detachment; and SUM is the total fit.

A typical PE spectrum is presented in Fig. 3, and shows several spectral contributions. At very low electron kinetic energy (eKE < 0.2 eV), a strong peak can be seen that decays exponentially with increasing eKE. Such features are typical for the statistical loss of electrons from hot ground state anions, *i.e.*, thermionic emission (TE). At the highest eKE in Fig. 3, a clear feature is present peaking at eKE = 2.0 eV (corresponding to an electron binding energy of 1.9 eV). This arises from some combination of direct detachment into the continuum and fast autodetachment from an excited resonance – both processes spectrally overlap. In our experiments, we cannot distinguish these processes so they are jointly labelled as prompt detachment (PD). Finally, there is an additional feature discernible between the TE and PD features. This feature appears not to shift significantly in eKE as the photon energy is scanned. In a similar vein to the interpretation of *p*BQ,^19^ we attribute this feature to electron ejection from a lower-lying resonance and label this process as delayed autodetachment (DA).

Fig. 2(a) reveals four main trends across the frequency-resolved spectra: (i) there is strong TE signal across the entire excitation range with notable intensity maxima around *hv* ∼ 2.0 eV and ∼3.0 eV; (ii) the PD spectral feature with eKE commensurate with *hv* (unit gradient) is intense in the 2.0 > *hv* > 2.75 eV and 3.5 > *hv* > 4.5 eV windows; (iii) a broad DA feature at the fixed eKE ∼ 0.25 eV, which is most clearly evident in the 3.0 > *hv* > 3.75 eV window; and (iv) for *hv* > 4.1 eV, a spectral feature commiserate with *hv* emerges at low eKE can be assigned to direct photodetachment that accesses neutral excited states, this is labelled as NE. Note that the *hv* = 3.10 eV PE spectrum has slightly reduced signal-to-noise compared with the other spectra because this wavelength corresponds to a change in the frequency mixing modes in the optical parametric oscillator.

Fig. 2(b) summarises a simultaneous (global) fit of all experimental PE spectra. The fitting procedure involves a number of assumptions: (i) the TE contribution is described by a single Richardson-like function^35^ with a temperature correlating with internal energy (*cf.*, increasing *hv*) assuming a harmonic vibrational partition function; (ii) the DA manifold assumes a Gaussian distribution convoluted with a Wigner threshold function near zero eKE (assuming *l* = 0);^36,37^ (iii) all spectral parameters such as the eKE centres and spectral widths of the DA, PD, and NE features are shared and constant across all PE spectra – with the exceptions that the NE and PD distribution centres in eKE space increase linearly with *hv* (*i.e.* fixed in electron binding energy). The final fit is that requiring the minimum number of basis functions that reproduces the experimental data in good accord without significant systematic unassigned residual (see ESI†). This fit provides a physically-meaningful deconvolution of the various detachment processes and, as we will show, is consistent with *ab initio* energetics and PE anisotropies. A representative PE spectrum at *hv* = 3.88 eV (320 nm) and model fit are shown in Fig. 3. This PE spectrum clearly illustrates the TE, DA, and PD features without the former dominating the spectrum, nor complications from the NE channels at higher *hv*.

The electron ejection angular distribution, quantified in terms of the conventional *β*_2_ parameter,^38^ are summarised in Fig. 2(c). Briefly, *β*_2_ ranges between –1 and 2, corresponding to electron ejection perpendicular or parallel to the laser polarization vector, respectively. The *β*_2_ values, given as a function of *hv* in Fig. 2(c), have been averaged over the centre of the relevant spectral feature. The angular distribution for TE, *β*TE2, remains approximately zero across all spectra, as might be expected for a slow thermal emission process. The anisotropy for DA, *β*DA2, is positive between 2.0 eV < *hv* < 2.7 eV, then suddenly changes to slightly negative for 2.8 eV < *hv* < 3.2 eV, and then becomes zero (isotropic) for *hv* > 3.2 eV. Finally, the anisotropy for PD, *β*PD2, gradually changes from positive at *hv* = 2.0 eV to strongly negative for *hv* > 2.7 eV. It should be stressed, however, that the DA and PD features spectrally overlap for *hv* < 2.5 eV, so the measured *β*_2_ magnitudes for *β*DA2 and *β*PD2 become difficult to interpret. In general, a non-zero *β*_2_ parameter implies electron ejection is occurring on a timescale faster than molecular rotation, and the sign of *β*_2_ can provide some qualitative information on the electronic character of the detachment processes involved.^19,39,40^ Specifically, in *p*BQ it was shown that abrupt changes in *β*_2_ as a function of excitation energy correlated with changes in electronic character of the resonances involved.^19^ This is evident in the menadione DA channel at around *hv* = 2.7 eV.

### Time-resolved PE spectroscopy

**Fig. 4:**
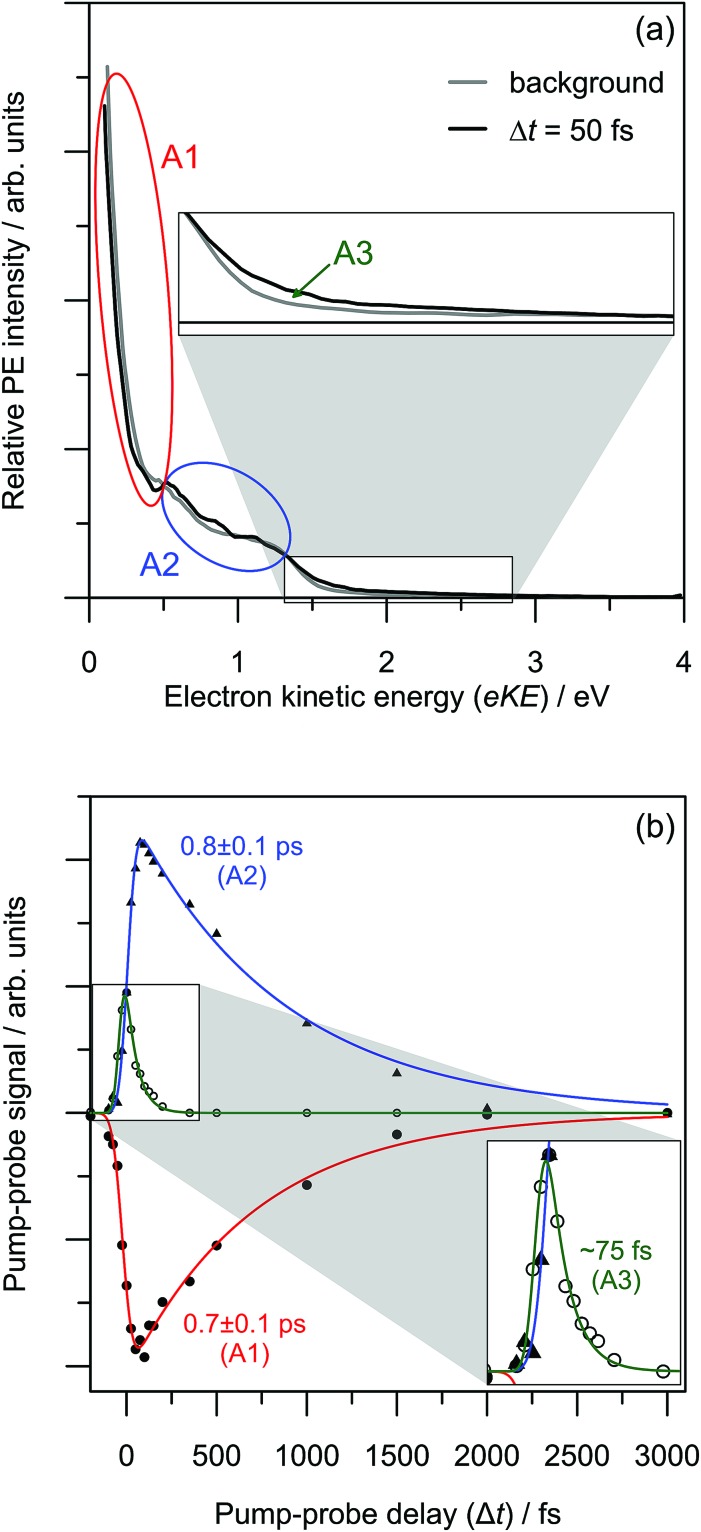
3.10 + 0.95 eV time-resolved photoelectron imaging: (a) a representative time-resolved photoelectron spectrum for Δ*t* = 50 fs (TE is truncated to emphasise the spectral changes). Three spectral features are identified, A1 (red), A2 (blue) and A3 (green); (b) relative populations of each pump-probe spectral feature as a function of pump-probe delay. Features A1 and A2 have a small degree of spectral overlap.

Results from the T-PI measurements using the 3.10 + 0.95 eV pump-probe scheme are summarised in Fig. 4. The pump photon is approximately resonant with photoexcitation to the 3^2^A′′ Feshbach resonance. As an example, the pump-probe PE spectrum for Δ*t* = 50 fs compared with the background (average of –3000 fs to –500 fs spectra) is given in Fig. 4(a). The series of Δ*t* spectra show three predominant changes: (i) a depletion in the thermionic contribution at low eKE; (ii) an increase in PE feature peaking at eKE ∼ 0.6 eV; and (iii) an increase in PE feature at higher eKE peaking at eKE ∼ 1.8 eV. The higher eKE feature is only present for Δ*t* within ∼100 fs of time zero.

To deconvolute the contributions of each pump-probe spectral feature, all background-subtracted pump-probe spectra were simultaneously fitted to a three-feature model. The model includes functions that describe: a thermionic emission profile (labelled A1); a skewed Gaussian distribution peaking at eKE ∼ 0.6 eV (labelled A2); and (iii) a Gaussian distribution centred around eKE ∼ 1.8 eV (labelled A3). Similar to the FA-PI modelling, all fitted distribution parameters are shared across all spectra and only the amplitudes are allowed to change across the Δ*t* series. The relative contributions of each spectral feature are summarised in Fig. 3(b). The resulting dynamic trend for A3 was fitted to an exponential decay convoluted with the instrumental cross-correlation, and those for A1 and A2 were fitted with convoluted growth and decay functions.

Feature A3 shows extremely fast dynamics and a lifetime of *τ*_A3_ ∼ 75 fs is extracted from the fit, although it is recognised that this is very close to the cross-correlation width of the pump and probe (70 fs). Feature A2, which peaks in the spectra at a lower eKE than A3, has only a small contribution at Δ*t* ∼ 0 fs, and its contribution reaches a maximum at Δ*t* ∼ 100 fs. The A2 decay lifetime is determined to be *τ*_A2_ = 0.8 ± 0.1 ps. Feature A1 has a negative area for Δ*t* > 0 fs, implying there is a net relative decrease in the spectral contribution of A1 in each PE spectrum compared with the background. The lifetime associated with the recovery of A1 was determined to be *τ*_A1_ = 0.7 ± 0.1 ps.

**Fig. 5:**
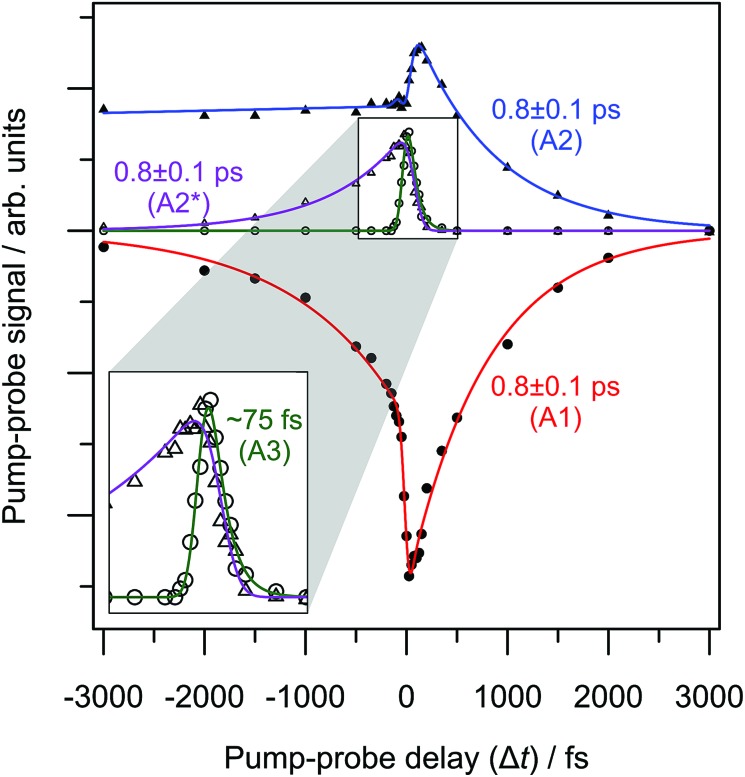
3.10 + 1.55 eV time-resolved photoelectron imaging: relative populations of each pump-probe spectral feature as a function of pump-probe delay, Δ*t*. Positive Δ*t* correspond to 3.10 eV pump and 1.55 eV probe. Feature A2* (purple) probes the dynamics of the same state as A2, but is excited directly and indirectly in negative and positive Δ*t*, respectively.

The 3.10 + 1.55 eV pump-probe scheme indicates time-resolved dynamics in both positive and negative Δ*t* directions, which are summarised in Fig. 5. Positive Δ*t* corresponds to a 3.10 eV pump while negative to a 1.55 eV pump. The +3000 fs PE spectrum was used as background. When the femtosecond temporal profiles overlap, both pump-probe sequences contribute to the time-resolved PE spectra. Fortunately, the spectral contributions of the time-varying features are sufficiently well-resolved to be disentangled (see ESI†). The full T-PI data series was amenable to a similar fitting procedure to that considered with the 3.10 + 0.95 eV data series.

In the 3.10 + 1.55 eV time-resolved PE spectra, the feature due to TE (A1) shows depletion and recovery in both temporal directions. Features A2 and A3 are present in the positive Δ*t* direction but shifted to higher eKE by 0.6 eV compared to the 3.10 + 0.95 eV data series (as expected from the increased probe energy). The fact that dynamics are observed for negative Δ*t* implies that at *hv* = 1.55 eV, an excited state of the menadione anion is accessed. For negative Δ*t*, an additional feature can be discerned, A2*. The extracted population dynamics are summarised in Fig. 5, along with fitted lifetimes.

In the positive Δ*t* direction, the same dynamics are observed for both probe energies: using a 1.55 eV probe yields lifetimes for the decay of the peak assigned to A2 as *τ*_A2_ = 0.8 ± 0.1 ps, while the TE emission (A1) recovers with a timescale of *τ*_A1_ = 0.8 ± 0.1 ps. The rapidly decaying feature initially excited at 3.10 eV (A3) has no negative component and arises with the cross-correlation. For negative time-delays, the additional feature, labelled as A2*, is also seen to decay on a timescale *τ*_A2*_ = 0.8 ± 0.1 ps. The decay of A2* is mirrored by a recovery of the TE feature in negative time.

Note that the contribution of A2 for negative Δ*t* levels-out to a constant value. This is because the pump-probe PE signal at each Δ*t* was determined using the same background PE spectrum of +3000 fs. That is, the 1.55 eV PE spectrum exhibits a different two-photon background over the A2 spectral region.

Finally, it is important to note that the integral PE signal in our pump-probe images was essentially constant at all delays. Hence, only by performing differential measurements, such as done in T-PI, can these dynamics be observed.

### *Ab initio* calculations

**Fig. 6:**
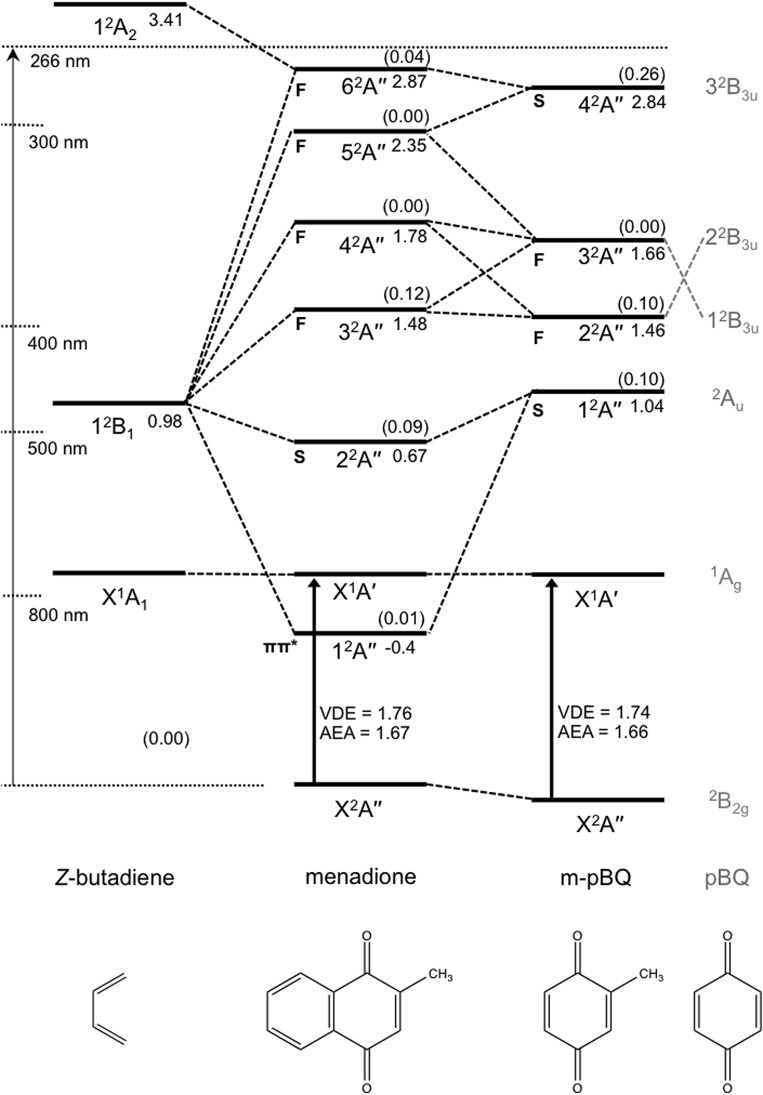
Energy level and correlation diagram of calculated electron excitation and detachment energetics for *Z*-butadiene, menadione, and m-*p*BQ. For m-*p*BQ, the corresponding resonances in *p*BQ are noted in grey. ‘S’ and ‘F’ indicate shape and Feshbach resonances, respectively. Calculated dipole transition oscillator strengths are given in parentheses. VDE is the vertical detachment energy and AEA is the adiabatic electron affinity. All energies are given in units of eV, and uncertainty in the calculated energetics are estimated at ±0.15 eV.

Results of the *ab initio* calculations are summarised in Fig. 6. Each resonance has been characterised by its predominant character: either a shape ‘S’ resonance involving predominately a HOMO → π* excitation; or a Feshbach ‘F’ resonance involving predominately a more inner-valence to π* transition (core-excited) and is energetically situated below its respective neutral state.^41^ Of particular note is a bound state of the anion calculated at ∼1.3 eV (vertically) above the anion ground state, denoted 1^2^A′′, and corresponds to a π → π* transition.

For comparison purposes, calculated energies for m-*p*BQ as well as *Z*-butadiene are also shown. Menadione can be viewed as being comprised of these two subunits and correlations between their states allows for a clear understanding of how the orbitals of menadione relate to and deviate from *p*BQ.^18^

Also included in Fig. 6 in parentheses are the calculated photoexcitation oscillator strengths. These oscillator strengths show that, for menadione, the shape resonance and lowest Feshbach resonance have non-zero oscillator strengths and are of similar magnitude to the energetically correlating resonances in m-*p*BQ and *p*BQ.^18,19^ One primary difference between menadione and either m-*p*BQ or *p*BQ within the FAT-PI window is that the resonances energetically analogous with the *p*BQ 3^2^B_3u_ state have significantly reduced excitation oscillator strength and have changed electronic character.

## Discussion

### Decay mechanisms of anion resonances

The FAT-PI results combined with the *ab initio* calculations indicate that menadione exhibits a number of optically-bright anion resonances leading to temporary anions, and that these are coupled very efficiently to the ground electronic state (X^2^A′′), either directly or sequentially *via* intermediate electronic states.

**Fig. 7:**
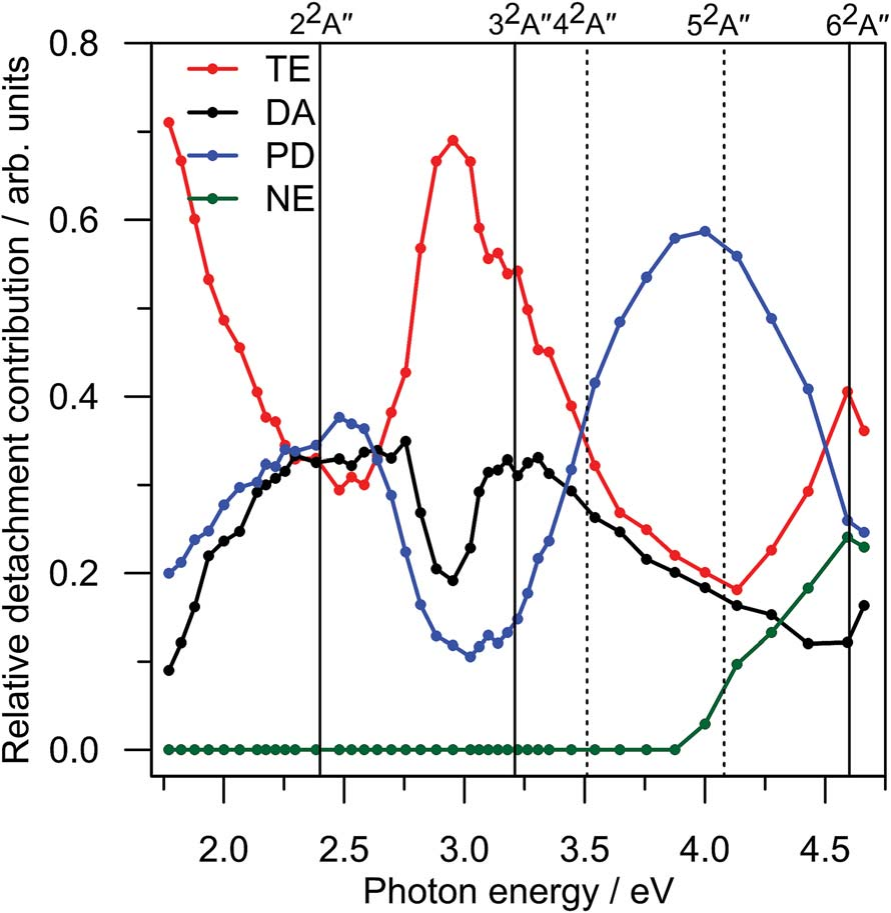
Relative contributions of each electron detachment process in the frequency-resolved PE spectrum (Fig. 2(b)). Vertical lines indicate the locations of excited state resonances (Fig. 6); solid lines are optically-active and dashed lines are optically-dark. TE corresponds to thermionic emission; DA to delayed autodetachment; PD to prompt detachment; and NE to detachment to neutral excited state(s). The TE contribution represents only a small proportion of that actually occurring. See further details in the text.

The trends identified across the FA-PI spectra result from the interplay between different electron detachment dynamics and electronic relaxation processes following photoexcitation. The contributions of each detachment channel from the fit shown in Fig. 2(b) are summarised in Fig. 7. Note that for *hv* < 2.3 eV, it is difficult for the fitting procedure to reliably deconvolute contributions from each channel.

The prompt detachment (PD) channel contains two contributions, photoexcitation directly into the detachment continuum and very fast (*i.e.* <10's fs) autodetachment. As both processes occur on timescales that are generally faster than nuclear rearrangement, the PD spectra arise essentially from a vertical transition and are determined by the Franck–Condon overlap between anion and neutral wavefunctions in the X^2^A′′ geometry. Additionally, a non-zero *β*_2_ might be expected. From the centre of the PD feature, eKE^PD^, the experimental vertical detachment energy (VDE) can be determined: VDE = *hv* – eKE^PD^. Using a linear extrapolation across all frequency-resolved PE spectra, the VDE was determined to be 1.89(2) eV.

Similarly, the adiabatic electron affinity (AEA) was determined to be 1.63(6) eV. There have been two other reports of electron affinities for menadione at 1.77(6) eV and 1.75(5) eV,^42,43^ However, it is unclear if these data derived from ion-molecule equilibria represent true adiabatic values. The calculated values were VDE = 1.76 eV and AEA = 1.67 eV, which are both within 0.15 eV of our experimental values.

The dominant feature in Fig. 2 is the strong thermionic emission (TE) feature. TE corresponds to statistical electron ejection from vibrationally-hot X^2^A′′ anion.^35,44–49^ In the case of menadione, the TE spectral contribution is present at all *hv* and is the dominant electron ejection process. Given that the experimental PE detector (MCP) gate width in the FA-PI acquisition only captures a small fraction of the TE electrons, the true relative contribution of TE in Fig. 7 will be much larger.‡
‡As a first estimation, assuming the MCP gate is of a well-defined trapezoid shape (DEI PVX-4140 ‘push–pull’ type pulsing unit) and also assuming that thermionic emission follows a mono-exponential profile with a 10–100 μs lifetime, then we expect to be measuring around a few percent of the total thermionic emission. However, because it is difficult to characterise fast pulses on capacitive-coupled elements (MCPs) operating under load, we cannot quantify this contribution with a high degree of certainty. The dominating contribution of TE across all PE spectra leads to three conclusions: (i) there is broad photoexcitation probability compared to non-resonant direct photodetachment across the entire *hv* range studied; (ii) nascent population of excited state resonances undergoes efficient internal conversion to the electronic ground state anion; and (iii) delayed autodetachment (DA) processes from resonances across the *hv* range are inefficient compared with internal conversion.

From Fig. 7, the relative PD contribution exhibits two broad maximum regions, centred at *hv* ∼ 2.4 eV and ∼4.0 eV. Interestingly, these two PD maxima correlate with minima in the TE contribution, suggesting that photoexcitation at these energies has a less efficient route to forming the ground state anion. Nevertheless, the fact that a large amount of TE (Fig. 2(a)) is observed at these energies suggests that there is still excitation to some resonances. Unfortunately, we have not been able to measure relative total PE cross-sections over the spectral window probed, however, we experimentally observed strong PE signal across the entire *hv* range. Total PE cross-section measurements would assist in assigning the location of resonances. Because such measurements are not available, we have turned to calculations that are discussed below.

The delayed autodetachment (DA) feature corresponds to spontaneous electron ejection from one or more quasi-bound resonances that become sequentially populated following internal conversion of the initially excited resonance. The FA-PI spectra indicate only one clear DA feature, which energetically correlates with electron ejection from the 2^2^A′′ shape resonance. Hence, the DA feature is assigned to autodetachment from the 2^2^A′′ shape resonance. Based on simple Koopmans theorem arguments, shape resonances might be expected to exhibit faster autodetachment rates than Feshbach resonances by virtue of minimal electronic reconfiguration to achieve the neutral state. The fact that the PE spectra do not show evidence of any energetically higher-lying DA features is consistent with the result that no higher-lying shape resonances were calculated, and suggests that autodetachment is only able to compete with internal conversion for the 2^2^A′′ shape resonance. Note that the overall yield of DA is still very small compared to TE, so internal conversion is still the predominant decay channel.

The DA contribution in Fig. 7 broadly indicates two maxima that energetically (in terms of photoexcitation) correlate with the calculated 2^2^A′′ shape and 3^2^A′′ Feshbach resonances, respectively. Based on the *ab initio* calculations, this agreement might be expected because these two resonances are those with non-zero optical oscillator strengths. For *hv* < 2.3 eV, DA and PD spectrally overlap so their relative contributions are somewhat tentative. The maximum in DA at around *hv* = 3.2 eV (where DA and PD do not spectrally overlap) indicates that photoexcitation to the 3^2^A′′ Feshbach resonance yields a small quantity of DA from the 2^2^A′′ shape resonance as well as a strong increase in the relative TE contribution.

The bimodal DA trend in Fig. 7, which has been assigned to the two optically-active resonances, is further supported considering the *β*_2_ parameters: *β*DA2 over the 2^2^A′′ photoexcitation region is different to that over the 3^2^A′′ photoexcitation region. For *hv* < 2.3 eV, it should also be recognised that the small degree of autodetachment from the 2^2^A′′ shape resonance might actually be by definition considered as PD rather than DA. In any case, the sudden change in *β*DA2 at *hv* ∼ 2.8 eV is suggestive of a change in mechanism leading to the electron detachment contribution in the DA manifold. That is to say, for *hv* < 2.8 eV, excitation populates the 2^2^A′′ shape resonance, while for *hv* > 2.8 eV, excitation populates the 3^2^A′′ Feshbach resonance. So, even though the orbital from which the electron is detached is the same, the initial excitation leads to different molecular frame ensemble alignment, which is reflected in the measured anisotropy. Similar trends in *β*DA2 have also been observed in *p*BQ,^19^ although the higher molecular point symmetry (D_2h_) resulted in larger *β*_2_ values between the analogous ^2^A_u_ shape and ^2^B_2u_ Feshbach resonances. The present discussion of *β*_2_ anisotropy is rather qualitative; however, a comprehensive theoretical framework to understand and model both instantaneous and delayed electron ejection anisotropy is a very difficult task.^50^ Nevertheless, angle-resolved PE imaging does provide useful additional information on sudden changes in the above-threshold dynamics.

The final contribution plotted in Fig. 7 is that from direct electron detachment to neutral excited (NE) electronic states. This channel is open for *hv* > 4 eV and, indeed, our *ab initio* calculations confirm the first neutral excited state is situated at ∼3.9 eV vertically above X^2^A′′. The NE feature spectrally overlaps with both the TE and DA contributions, meaning that the other fitted channel contributions in Fig. 5 become less reliable for *hv* > 4 eV. Specifically, of the higher-lying resonances, 4^2^A′′, 5^2^A′′, and 6^2^A′′, only the latter has a small but non-zero calculated oscillator strength. While there is evidence for TE contaminant with photoexcitation resonant with the 6^2^A′′, this contribution is difficult to resolve from the NE channel.

The 3.10 + 0.95 eV time-resolved measurements are in complete accord with the above picture, but provide additional insight into the decay pathways and mechanism from the 3^2^A′′ resonance. Feature A3 can be spectrally assigned to a combination of the initially excited 3^2^A′′ Feshbach resonance and DA from the 2^2^A′′ shape resonance. Hence, we also assign the time evolution of the A3 population to both these resonances. Unfortunately, we were unable to resolve the dynamics of internal conversion from the 3^2^A′′ to the 2^2^A′′ resonance, and therefore cannot definitively conclude that the 3^2^A′′ state decays only to the 2^2^A′′ resonance; there may be some competing internal conversion pathways. Regardless, the ∼75 fs lifetime of A3 indicates that both the 3^2^A′′ and 2^2^A′′ resonances have decayed on this timescale, predominantly by internal conversion.

As feature A3 decays, feature A2 grows in, suggesting that population flows from the 3^2^A′′ and 2^2^A′′ resonances into a state producing the PE feature associated with A2. The A2 feature can be assigned to the photodetachment from the 1^2^A′′ valence-bound state; the mean eKE at ∼0.6 eV matches that expected from the *ab initio* energies with a 0.95 eV probe. The population of the 1^2^A′′ state subsequently decays with a lifetime of 0.8 ± 0.1 ps. This decay is mirrored by the recovery of the TE emission feature (A1), indicating that the mechanism associated with the decay of the 1^2^A′′ bound state is internal conversion to form the X^2^A′′ ground state anion.

The 3.10 + 1.55 eV time-resolved PE spectra are consistent with the above dynamics when the 3.10 eV photon acts as the pump (positive Δ*t*); the highest eKE feature corresponding to the 3^2^A′′ and 2^2^A′′ resonance population similarly decays on a ∼75 fs timescale, while the feature that can be assigned to the 1^2^A′′ bound state decays on a 0.8 ± 0.1 ps with a concomitant recovery of the TE feature.

In the negative Δ*t* direction, the 1.55 eV pump can resonantly access the 1^2^A′′ bound state directly as it has a weak but sufficient oscillator strength. The relative intensities in the positive and negative Δ*t* direction are different due to a combination of different pump laser fluence and photoexcitation cross-sections, as well as probe photodetachment cross-sections between the two pumped states. Nevertheless, a feature in the time-resolved PE spectra for negative Δ*t* can be discerned (A2*) that spectrally corresponds to the detachment from the 1^2^A′′ bound state. The decay profile of A2* in Fig. 5 indicates that the lifetime of the 1^2^A′′ bound state is 0.8 ± 0.1 ps and decays by internal conversion because the TE recovery mirrors this decay. Overall, the assignment in the 3.10 + 0.95 eV pump-probe experiments of feature A2 to the 1^2^A′′ bound state is consistent with the 1.55 + 3.10 eV T-PI spectra, in which the dynamics of the 1^2^A′′ bound state are probed directly.

The lowest quartet anion state is situated 4.1 and 3.6 eV above the ground state anion in ground state doublet and excited quartet state geometries. We can therefore discount any involvement of quartet states in the observed pump-probe dynamics.

**Fig. 8:**
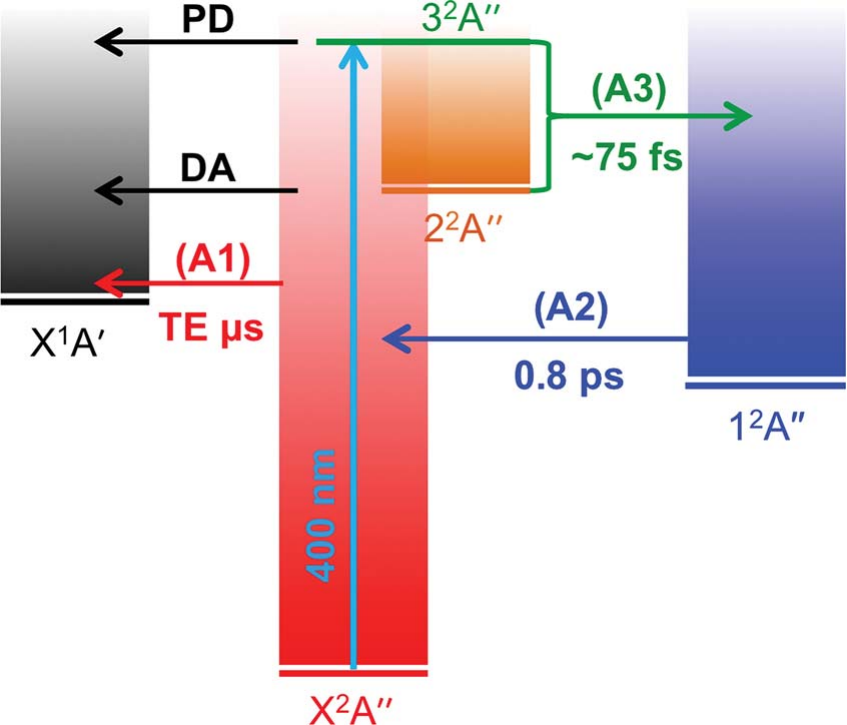
Schematic summary of menadione temporary anion dynamics following absorption of a 3.10 eV (400 nm) photon. A1, A2, and A3 are the three pump-probe features identified in the time-resolved imaging experiments, and DA is the small contribution of delayed autodetachment identified in the frequency- and angle-resolved imaging experiments. TE corresponds to thermionic emission, which is the predominant electron loss channel.

Based on the data presented above, a detailed account of the above-threshold dynamics of the menadione radical anion emerges, which is captured schematically in Fig. 8. Initial population of resonances up to 1.4 eV above threshold undergo rapid internal conversion to form the bound valence 1^2^A′′ state, with autodetachment being a very minor, but open, channel. Electronic relaxation processes involve conical intersection seams between the various states, and their efficiency is likely enhanced by the high density of vibronic states.^51–53^ Optical preparation of the 2^2^A′′ and 3^2^A′′ resonances exhibit broad excitation profiles, and additional resonances (4^2^A′′, 5^2^A′′, and 6^2^A′′) at higher energies (up to 3.0 eV above the neutral probed here) undergo similar decay paths as evidenced by the fact that a strong TE contribution can be observed across the entire *hv* range. The strong TE contribution also indicates that the direct photodetachment cross-section into the neutral ionisation continuum is relatively small. In the case of menadione, the existence of the 1^2^A′′ valence-bound excited state certainly plays an important role as an intermediate in coupling resonances to the ground electronic state. Although the lifetime of the 1^2^A′′ state is relatively long, autodetachment is inhibited as it is vertically bound and rapid internal vibrational relaxation will redistribute the internal energy. Once the 1^2^A′′ state has decayed by internal conversion, the ground state anion is reformed. In the isolated gas-phase environment, where no collision can remove the excess internal energy, the system decays by TE over a long (microsecond) timescale.

### Effect of molecular size, delocalization, and density of states

In some respects, menadione can be viewed as an extension of *p*BQ because both species exhibit analogous resonances that may capture free electrons in a similar sequence of events.^18,19^ Use of the term ‘extension’ is used rather than saying that *p*BQ is the electrophore in menadione, because the active resonances in menadione are strongly delocalised over the *Z*-butadiene entity. Actually, there are two key differences. First, photoexcitation to prepare the 3^2^A′′ resonance in menadione (predominantly orbital 6 → 9 in the ESI†) involves migrating electron density from the *Z*-butadiene subunit to the m-*p*BQ subunit. Second, photoexcitation to populate either of the 1^2^A′′ or 2^2^A′′ states (predominantly orbitals 9 → 10 and 9 → 11 in the ESI,† respectively) involve excitation from an orbital localised on the m-*p*BQ subunit to orbitals delocalised over the entire molecule. That is, addition of the conjugated *Z*-butadiene unit to m-*p*BQ plays an active role in electron delocalisation of these anion resonances, and thus implies they have drastically changed in character compared with the analogous resonances in *p*BQ.

The ultrafast dynamics of menadione and *p*BQ anions also show some similarities and differences.^18,19^ In *p*BQ, initial excitation to the 2^2^B_3u_ Feshbach resonance led to DA from the lower-lying ^2^A_u_ shape resonance, which was populated following rapid internal conversion on a ∼20 fs timescale. Excited state *ab initio* calculations revealed that a conical intersection between the anionic 2^2^B_3u_ and ^2^A_u_ resonances was responsible for the efficient coupling. The electronic relaxation processes are overall quite similar in menadione. However, there are also very important differences. Specifically, although the 3^2^A′′ → 2^2^A′′ internal conversion in menadione anions may be viewed as analogous to the 2^2^B_3u_ → ^2^A_u_ internal conversion in *p*BQ anions, the subsequent internal conversion rates are very different. For temporary *p*BQ anions, the dominant decay mechanism from the ^2^A_u_ state is autodetachment; even though TE was observed, this was significantly weaker than DA. In anionic menadione, TE dominates the PE spectra at all *hv*. The route to forming the ground state, which is unavailable to *p*BQ anions, is relaxation to the 1^2^A′′ bound excited state. This low-lying excited state comes about from a combination of the π-system of *Z*-butadiene and m-*p*BQ as shown in Fig. 5. Thus, it is by virtue of the extended and conjugated π-system that for the larger delocalised molecular systems, there are lower-lying states that can aid in funnelling quasi-bound population from above-threshold into bound states. Additionally, the increased vibrational state density for menadione compared with *p*BQ is likely to enhance the internal conversion coupling probabilities between various excited states.^53^ Further, it cannot be ruled out that an increase in vibrational state density could also decrease the rate of autodetachment. For *p*BQ anions, there are also some lower-lying states of nπ* character, however these were apparently less efficient in directing population from the ππ* resonances to the ground state. Analogous nπ* resonances in menadione anions are not within the optical photoexcitation window considered in the present study.

Although the resonance energetics and oscillator strengths (assuming anion geometries) within the FA-PI *hv* window are similar in both menadione and *p*BQ anions for the two lowest optically-active resonances, they differ for the higher resonances. Specifically, the 3^2^B_3u_ shape resonance in *p*BQ has a large oscillator strength compared with the other resonances, and earlier total PE cross-sections indicate a large relative cross-section to photodetachment.^54^ For *p*BQ anions, the 3^2^B_3u_ resonance decayed primarily by autodetachment (*i.e.* PD dominated) and there was no evidence for internal conversion and TE.^19^ In menadione, the energetically analogous resonances have predominant Feshbach character and have decreased oscillator strengths. Thus, it is expected that these higher resonances will have lower probabilities of being optically excited. Additionally, Feshbach resonances typically have longer autodetachment lifetimes, which, combined with the higher density of lower lying states in menadione anions, means that internal conversion outcompetes autodetachment and DA. This is evidenced by the fact that the menadione PE spectra show TE even at these higher photon energies.

A number of low-energy electron attachment experiments by Pshenichnyuk and co-workers^28,29^ have identified long-lived anions across a series of naphthoquinone molecules, namely 9,10-naphthoquinone and twelve naphthoquinone derivatives. The first of these molecules differs from menadione by the single methyl group. Their studies are not sensitive to the detailed time-resolved dynamics nor the competition between detachment processes that have been identified in the present study, however, they were able to characterise some properties of TE that we were unable to measure. Specifically, the total detachment lifetime (dominated by TE) was on the order of tens to hundreds of microseconds and depends on the energy of the initially occupied resonance; higher-lying resonances produce hotter ground state anion, leading to shorter TE lifetimes. Naphthoquinone was the only species that formed long-lived anions from population of two resonances, while all other naphthoquinone-derivatives formed long-lived anions from population of three resonances, including 2-hydroxy-1,4-naphthoquinone, which has a hydroxyl group instead of the methyl group in menadione. Thus, it appears that the addition of one non-conjugated group on the quinone subunit can influence the production of ground state anions. The menadione CASSCF reference wavefunctions in the present study indicate that the orbital associated with the 5^2^A′′ and 6^2^A′′ resonances has a significant contribution from the carbon atom to which the methyl group (or hydroxyl in 2-hydroxy-1,4-naphthoquinone) are bonded. Hence, it is unsurprising that the identity of the substitution on the *p*BQ subunit could modify the internal conversion dynamics of the higher-lying resonances. Finally, an electron transmission study by Modelli and Burrow^55^ considering *p*BQ and 1,5-dimethyl-*p*BQ (*i.e.*, *p*BQ with two *ortho*-methyl groups) noted changes in electron capture cross-sections of the quasi-bound resonances. Specifically an increase in capture cross-section for the higher-lying resonance was observed for 1,5-dimethyl-*p*BQ, which further supports the suggestion that functional group alkylation modifies the electron capture dynamics on *p*BQ. Further consideration on the electronic effects of functional group substitution and changes in the temporary anion dynamics probed by FAT-PI will be the subject of a future study.

### Relevance to anion formation and electron transfer reactivity

Although elastic electron scattering cross sections are typically much larger than inelastic, our results demonstrate that when resonances in menadione are populated, the predominant outcome is the formation of the ground state anion. From an astrophysical perspective, there is an abundance of free electrons in space, and it is established that large carbon-rich and highly-delocalised species with a small number of oxygen and nitrogen atoms (*i.e.*, similar to menadione) are present in the interstellar medium.^3^ In this environment, the formation of ground state anions is not surprising given that similar efficient routes to forming them are likely to exist. However, as the interstellar medium is a non-interacting environment, the final energy must somehow be disposed of without the loss of the electron by TE. The TE lifetime then becomes of interest; if sufficiently long, black-body radiation from the system can compete as a process to dispose of the excess internal energy. This radiation may contribute to the infrared bands observed from the interstellar medium.

If some anions can be formed in a stable ground state through a sequence of steps similar to those for menadione, then they will still be subject to large amounts of, for example, UV radiation. For menadione radical anion, absorption of such radiation does not lead directly to electron loss but regenerates the ground state which is then subject to TE and black-body emission. From this perspective, it is reasonable to conclude that the formation and abundance of anions in the interstellar medium is likely to be closely linked to the available decay mechanism of anion resonances and the ultimate lifetime of TE. For larger molecular species with similar excited state resonances and a ground state electron affinity similar to menadione, an increase in the number of vibrational degrees of freedom should increase the average lifetime of ground electronic state anions,^53,56^ and decrease the probability of TE relative to thermal photoemission. Hence, stable larger (and conjugated) molecular anions are likely to be formed more readily.

**Fig. 9:**
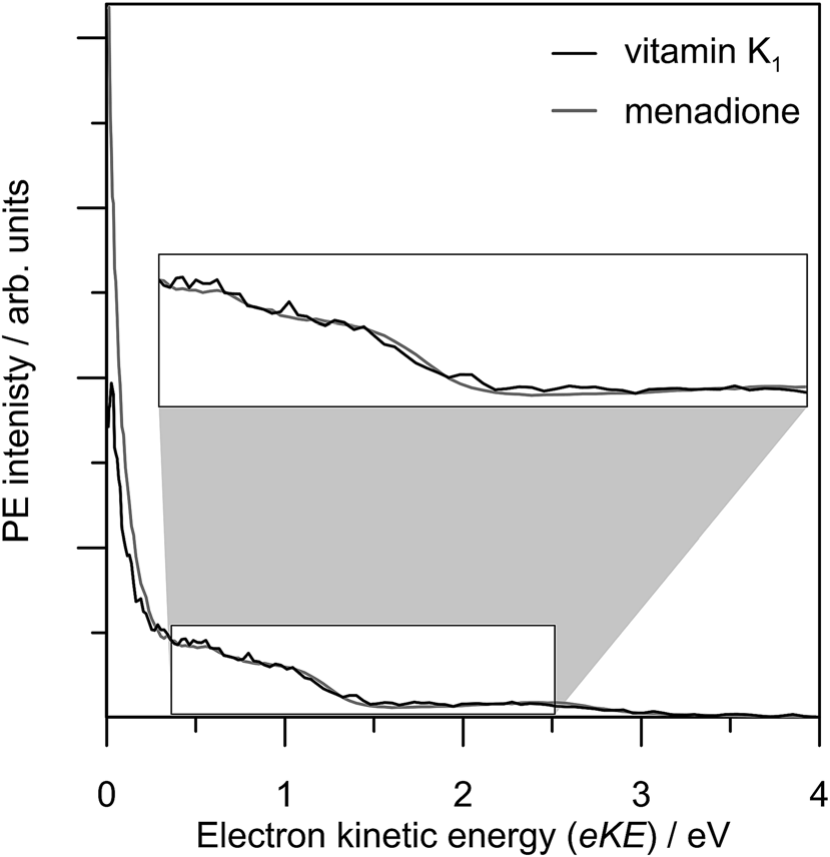
3.10 eV photoelectron spectrum of vitamin K_1_ (black line) compared with menadione (grey line).

From the biological perspective and concerning electron transfer, resonances in menadione can act as electron acceptor states which are, in turn, coupled in an ultrafast manner to the ground state. These dynamics may provide an efficient means to bypass a free energy barrier in Marcus' inverted region.^57–59^ Indeed, quinones are found widely throughout biological and technological charge-transfer complexes.^60–63^ We have measured a 3.10 eV (400 nm) femtosecond PE spectrum of vitamin K_1_, given in Fig. 9, and this is clearly very similar to that of menadione at the same photon energy. The disagreement between the TE contributions is due to a combination of operating the detector under different (non-linear) gain conditions for vitamin K_1_, and that this larger molecule may have an increased TE lifetime. Vitamin K_1_ is difficult to electrospray in a reasonable yield, so the comparison with menadione has only been considered at a single photon energy. Overall, it can be confidently stated that menadione is the electrophore in vitamins K_1_ and K_2_, and the non-conjugated side chain primary function is to enable miscibility in lipids.

The importance of low-lying excited states for electron transfer reactivity was recently pointed out by Liu and Troisi.^64^ In fullerenes, which are outstanding electron acceptors, the availability of low-lying (0.2–0.4 eV) excited states of their anions was shown to dramatically enhance electron transfer rates. In addition, the authors outlined a number of nitrogen- and/or oxygen-containing polyaromatic species that also have low-lying bound excited anion states, some of which are not all too structurally dissimilar to menadione. In menadione, the availability of the low-lying 1^2^A′′ state with a short lifetime will enhance the molecules ability as an electron acceptor.

## Conclusions

This paper has detailed frequency-, angle-, and time-resolved photoelectron imaging (FAT-PI) together with *ab initio* calculations to elucidate the dynamics of anion resonances leading to the formation of the ground state anion of the large prototype molecular radical anion, menadione. FAT-PI can provide a comprehensive understanding of temporary anion dynamics. For menadione, a number of optically-accessible anionic resonances have been identified, and it has been unambiguously demonstrated that anion resonances can act as doorway states to form metastable anions. Population of these resonances has an exceptionally high probability of being efficiently and rapidly internally-converted into the ground state anion, even when situated 3 eV above threshold. The vibrationally-hot ground state anion then undergoes statistical thermionic emission. Comparisons with *p*BQ and a series of electron attachment studies on naphthoquinone-derivatives suggests similar dynamics may be occurring in structurally similar species. However, an increase in the density of states in accord with molecular size and conjugation will allow more efficient pathways to funnel quasi-bound excess electron population towards the ground electronic state. It is expected that similar processes might occur in related polycyclic and delocalized species, and our results provide an important step towards understanding the mechanisms of anion formation in, for example, the interstellar medium. From an electron transfer perspective, menadione is the electrophore in the natural vitamin K species, which are known to be important biological electron transport mediators. The availability of excited states that can efficiently produce the ground state anion may provide convenient by-passes for the free energy barrier in the inverted region of charge-transfer.

From a methodological point-of-view, the above-threshold dynamics gleaned through FAT-PI are analogous to the dynamics of neutral electron capture assuming an understanding of differences in starting geometries and photoexcitation cross-sections *vs.* electron capture cross-sections. Geometry differences can be readily explored through *ab initio* calculations. Overall, FAT-PI is a powerful method to studying anion formation and anion resonance dynamics, and can be applied to any molecular species that can be electrosprayed as a stable electronic ground state anion.
