# Supplementary material for: On the formation of anions: frequency-, angle-, and time-resolved photoelectron imaging of the menadione radical anion
Source: Chem Sci. 2014 Dec 17;6(2):1578–89. doi: 10.1039/c4sc03491k (PMC5811081; doi:10.1039/c4sc03491k)

Electronic Supplementary Material (ESI) for Chemical Science.
This journal is © The Royal Society of Chemistry 2014

Electronic Supplementary Information (ESI) for 'On the formation of anions: Frequency-, angle-, and time-resolved photoelectron imaging of the menadione radical anion'

Electronic structure calculations

Menadione X^2A'' CASSCF geometry

ATOM	CHARGE	X	Y	Z
C	6.0	1.7361542005	2.5046150142	0.0000000000
C	6.0	0.3987207857	2.1536165314	0.0000000000
C	6.0	-0.0025898260	0.7908050780	0.0000000000
C	6.0	0.9905488703	-0.2125033071	0.0000000000
C	6.0	2.3586600963	0.1649254610	0.0000000000
C	6.0	2.7310191841	1.4965963127	0.0000000000
H	1.0	2.0238987606	3.5486928278	0.0000000000
H	1.0	-0.3728159808	2.9078339194	0.0000000000
H	1.0	3.0991685260	-0.6202612349	0.0000000000
H	1.0	3.7789768593	1.7695618999	0.0000000000
C	6.0	0.6241515892	-1.6372529160	0.0000000000
C	6.0	-1.4371597365	0.4610268762	0.0000000000
C	6.0	-1.7677755813	-0.9398589198	0.0000000000
C	6.0	-0.7803839891	-1.9122553597	0.0000000000
O	8.0	1.4921674998	-2.5300291298	0.0000000000
O	8.0	-2.2965046001	1.3658258979	0.0000000000
C	6.0	-3.2253689743	-1.3450516735	0.0000000000
H	1.0	-3.8657998996	-0.4667005675	0.0000000000
H	1.0	-3.4693538393	-1.9474711311	-0.8809290063
H	1.0	-3.4693538393	-1.9474711311	0.8809290063
H	1.0	-1.0610071054	-2.9596394479	0.0000000000

Menadione X^1A' CASSCF geometry

ATOM	CHARGE	X	Y	Z
C	6.0	1.7563512673	2.5142934310	0.0000000000
C	6.0	0.4080898147	2.1653182901	0.0000000000
C	6.0	0.0323800228	0.8123362338	0.0000000000
C	6.0	1.0201090211	-0.1834285956	0.0000000000
C	6.0	2.3773256922	0.1733611149	0.0000000000
C	6.0	2.7429954719	1.5169931940	0.0000000000
H	1.0	2.0416387961	3.5564721346	0.0000000000
H	1.0	-0.3590472714	2.9228678213	0.0000000000
H	1.0	3.1233543412	-0.6054052528	0.0000000000
H	1.0	3.7882286092	1.7907856257	0.0000000000
C	6.0	0.6226174764	-1.6181271304	0.0000000000
C	6.0	-1.4168988939	0.4503463960	0.0000000000
C	6.0	-1.7930071489	-0.9953271452	0.0000000000
C	6.0	-0.8206178388	-1.9351252759	0.0000000000
O	8.0	1.4452085372	-2.5097704538	0.0000000000

O	8.0	-2.2697912909	1.3117203919	0.0000000000
C	6.0	-3.2598214600	-1.3587826277	0.0000000000
H	1.0	-3.8818906331	-0.4695736983	0.0000000000
H	1.0	-3.5058506604	-1.9520395542	-0.8815797763
H	1.0	-3.5058506604	-1.9520395542	0.8815797763
H	1.0	-1.0601701921	-2.9898703453	0.0000000000

Optimized MCSCF orbitals in the menadione CASSCF active space

π and O(p) orbitals

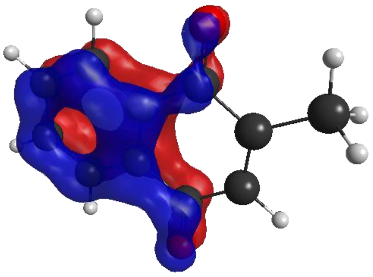

1

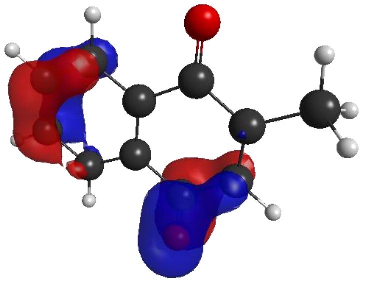

2

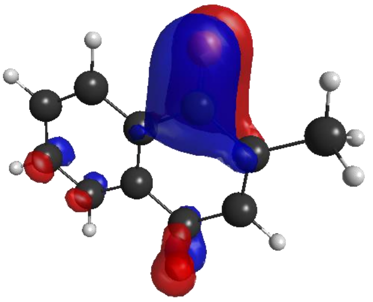

3

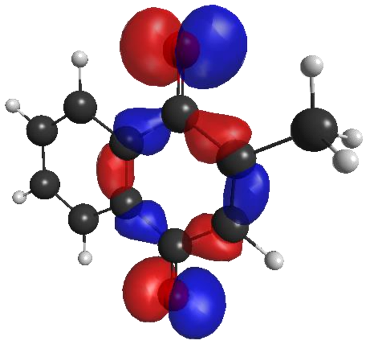

4 O(p)

π* orbitals

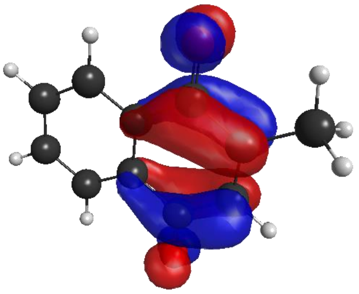

9

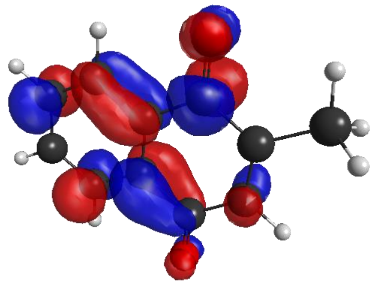

10

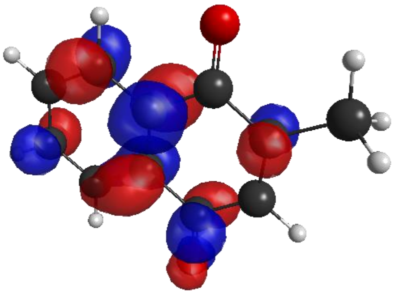

11

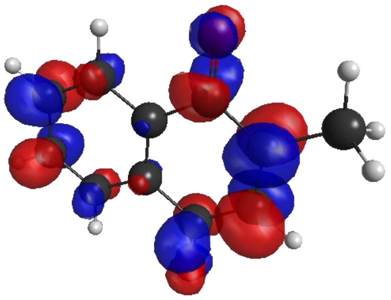

12

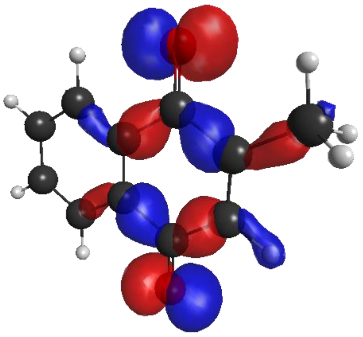

5 O(p)

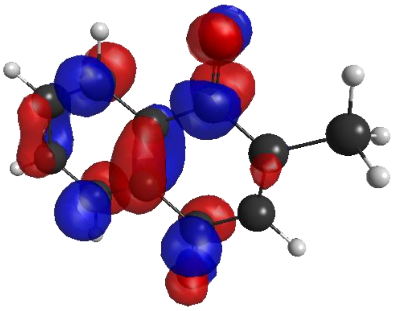

13

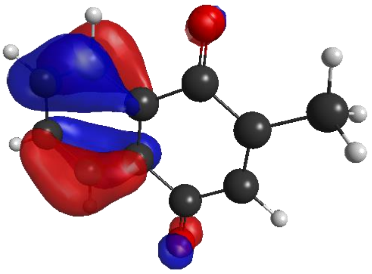

6

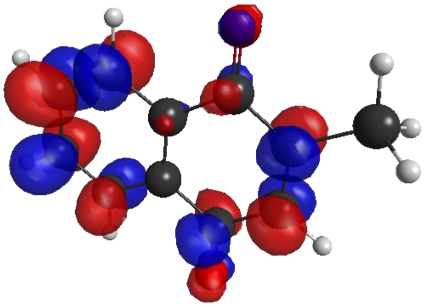

14

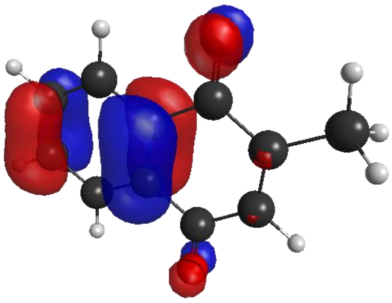

7

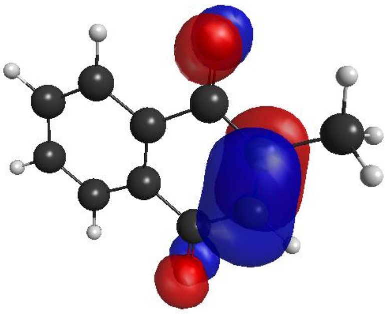

8

Residuals in the global frequency-resolved photoelectron spectrum fit

Residual is plotted on the same intensity scale as the simulated signal. The small residuals are proportional to the noise in each reconstructed photoelectron spectrum.

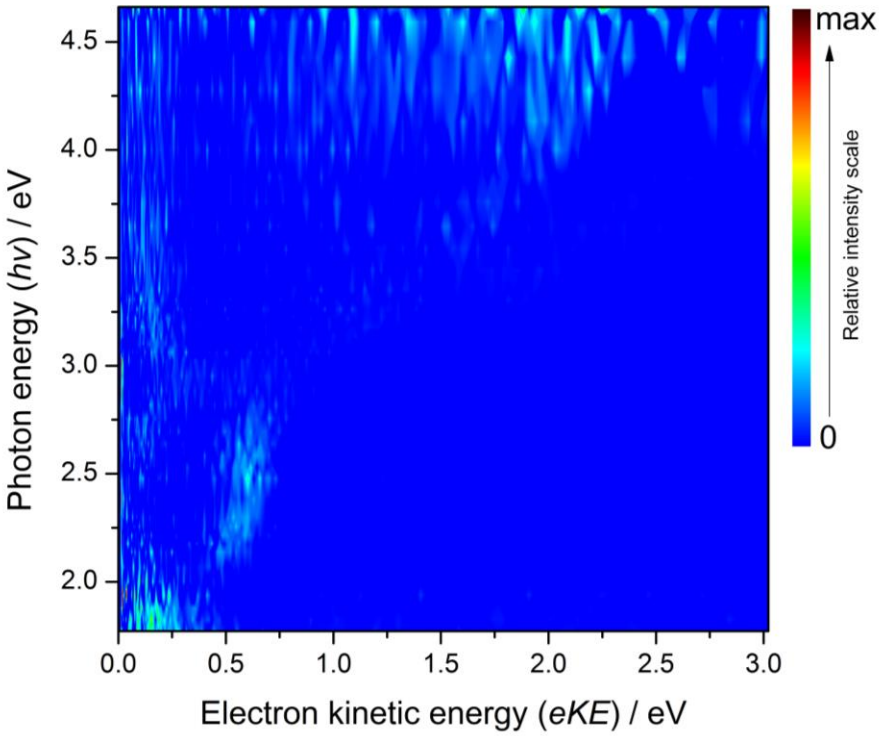

All photoelectron spectra included in the frequency-resolved plot

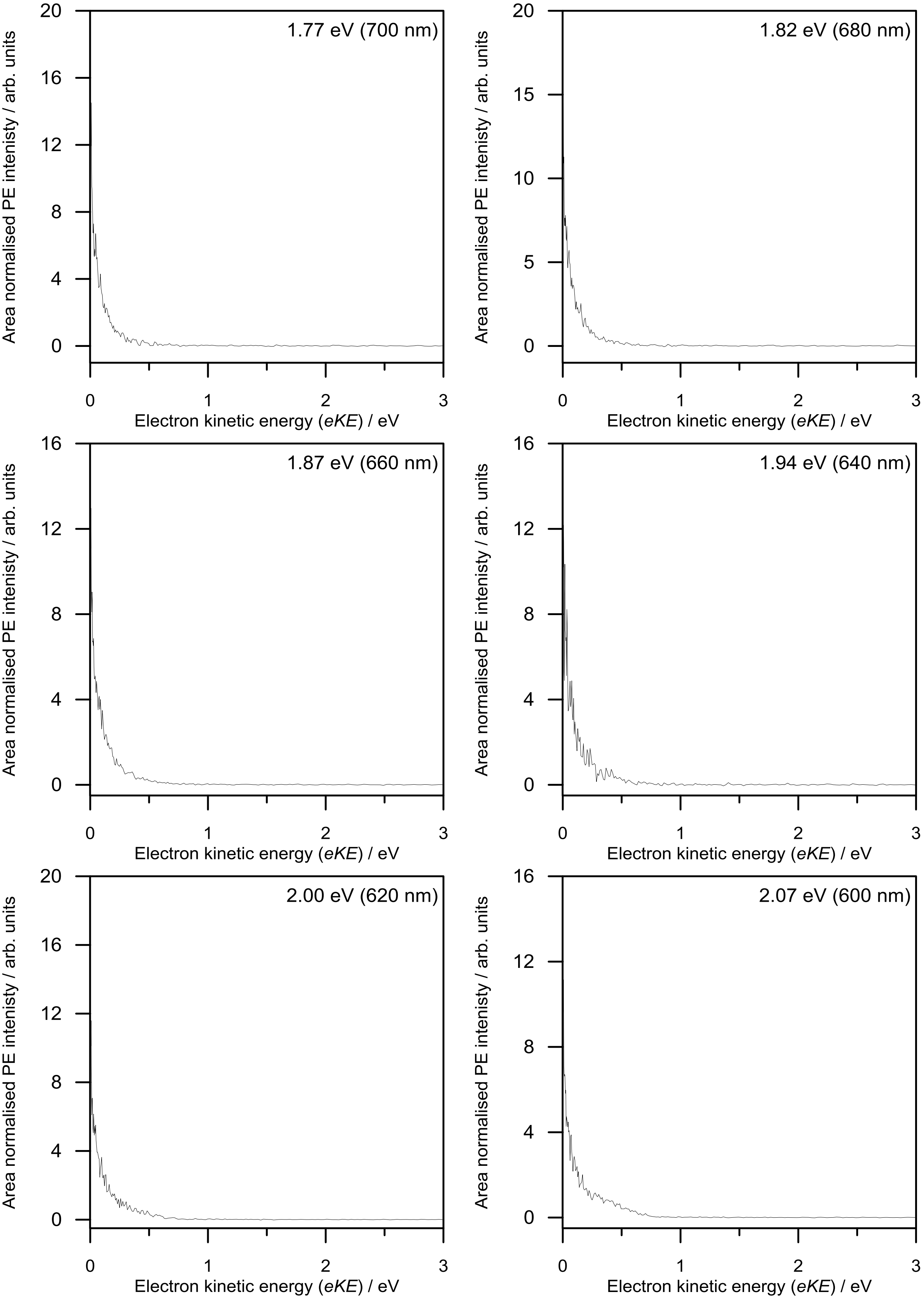

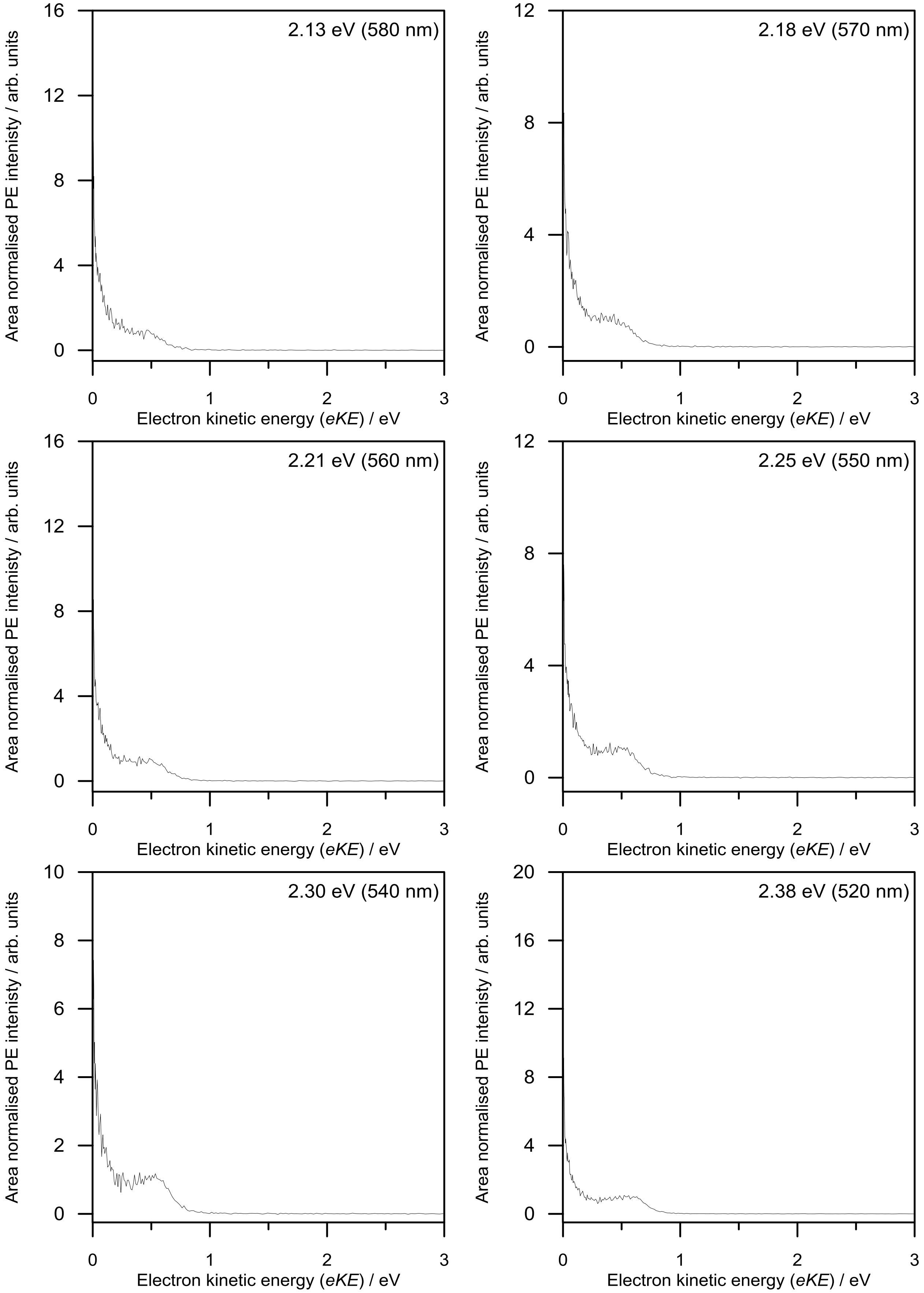
2.13 eV (580 nm)
2.18 eV (570 nm)
2.21 eV (560 nm)
2.25 eV (550 nm)
2.30 eV (540 nm)
2.38 eV (520 nm)
Area normalised PE intenisty / arb. units
Electron kinetic energy (eKE) / eV
0
1
2
3
0
4
8
12
16
20
2
6
10

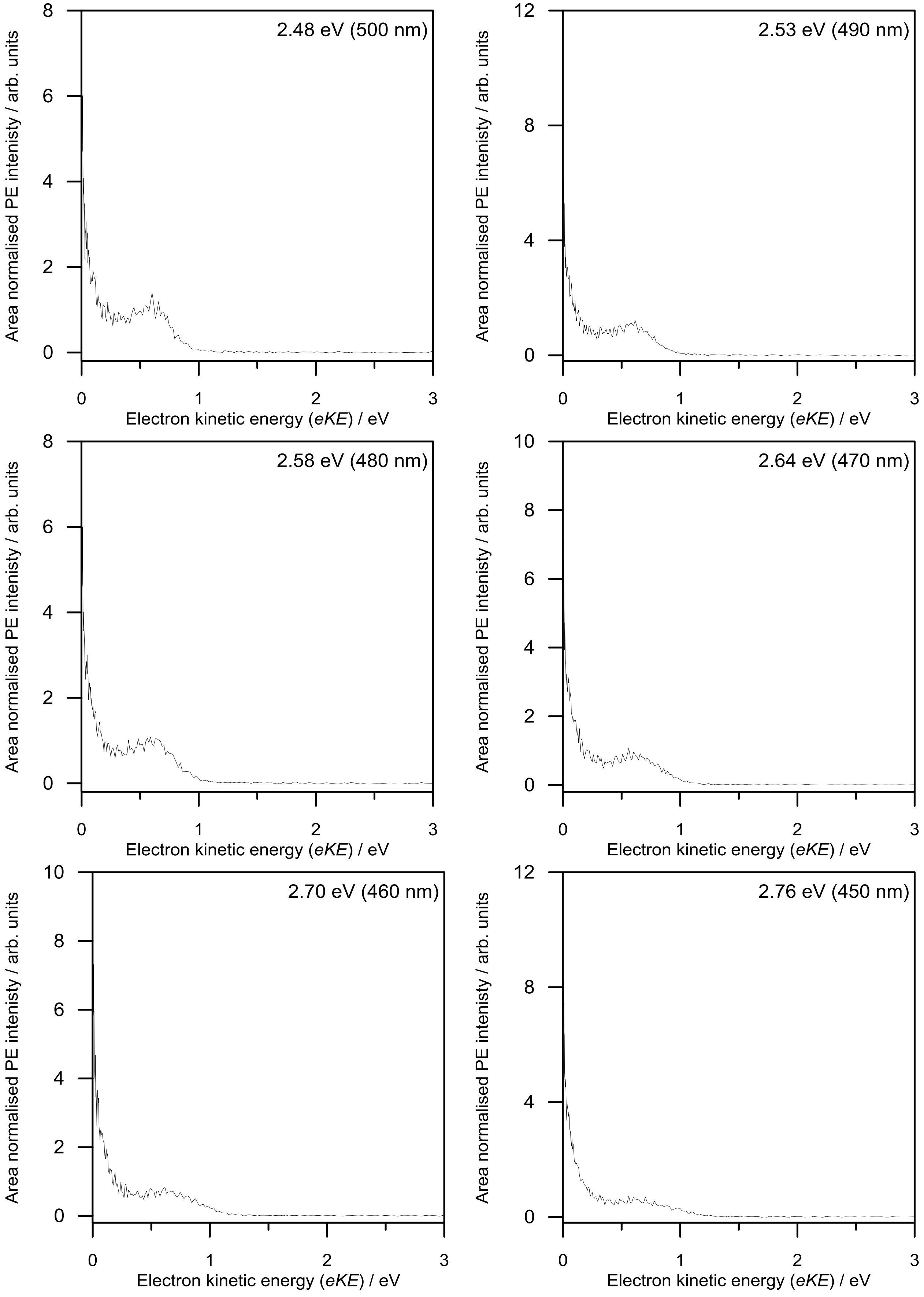

2.48 eV (500 nm)
2.53 eV (490 nm)
2.58 eV (480 nm)
2.64 eV (470 nm)
2.70 eV (460 nm)
2.76 eV (450 nm)
Area normalised PE intenisty / arb. units
Electron kinetic energy (eKE) / eV

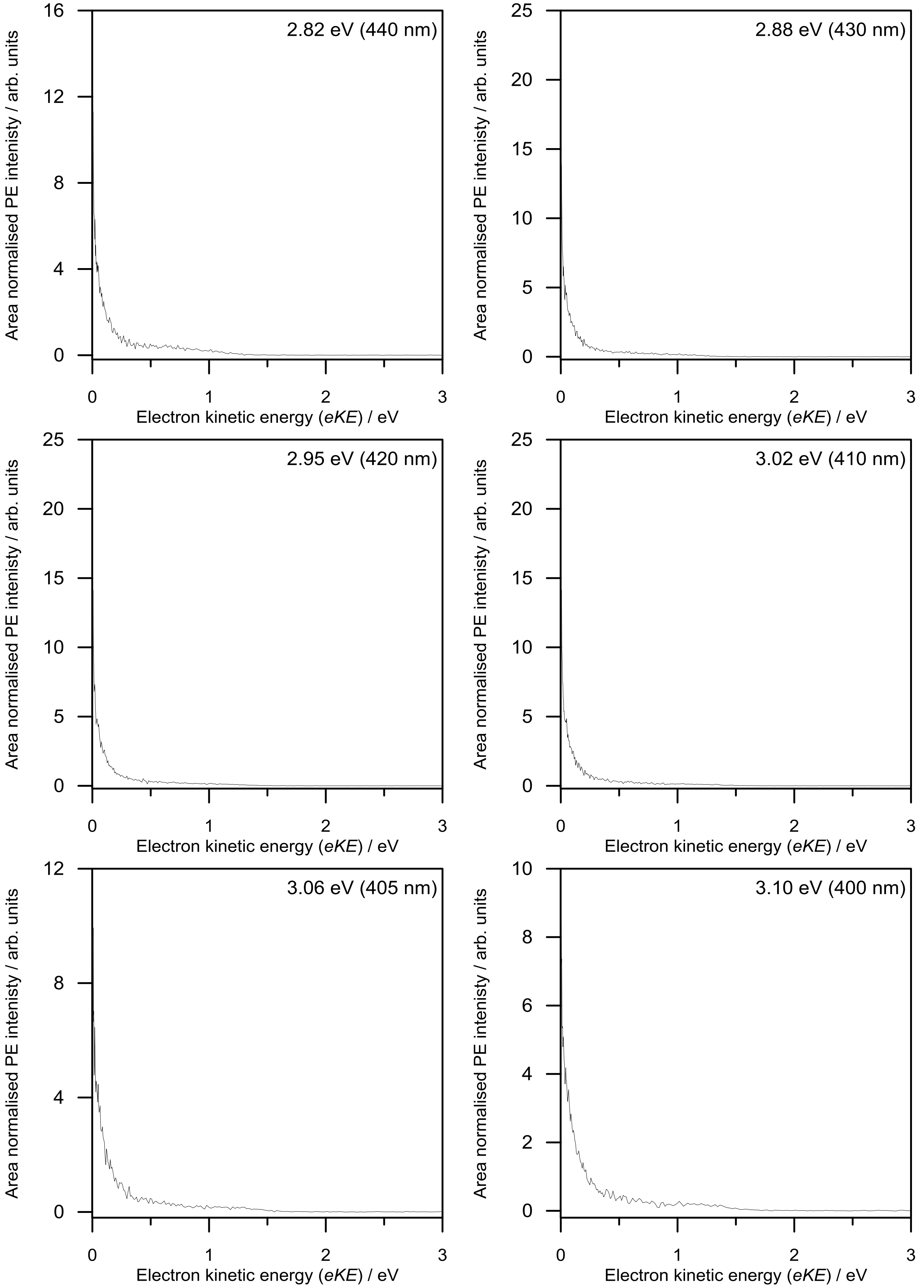
2.82 eV (440 nm)
2.88 eV (430 nm)
2.95 eV (420 nm)
3.02 eV (410 nm)
3.06 eV (405 nm)
3.10 eV (400 nm)
Area normalised PE intenisty / arb. units
Electron kinetic energy (eKE) / eV

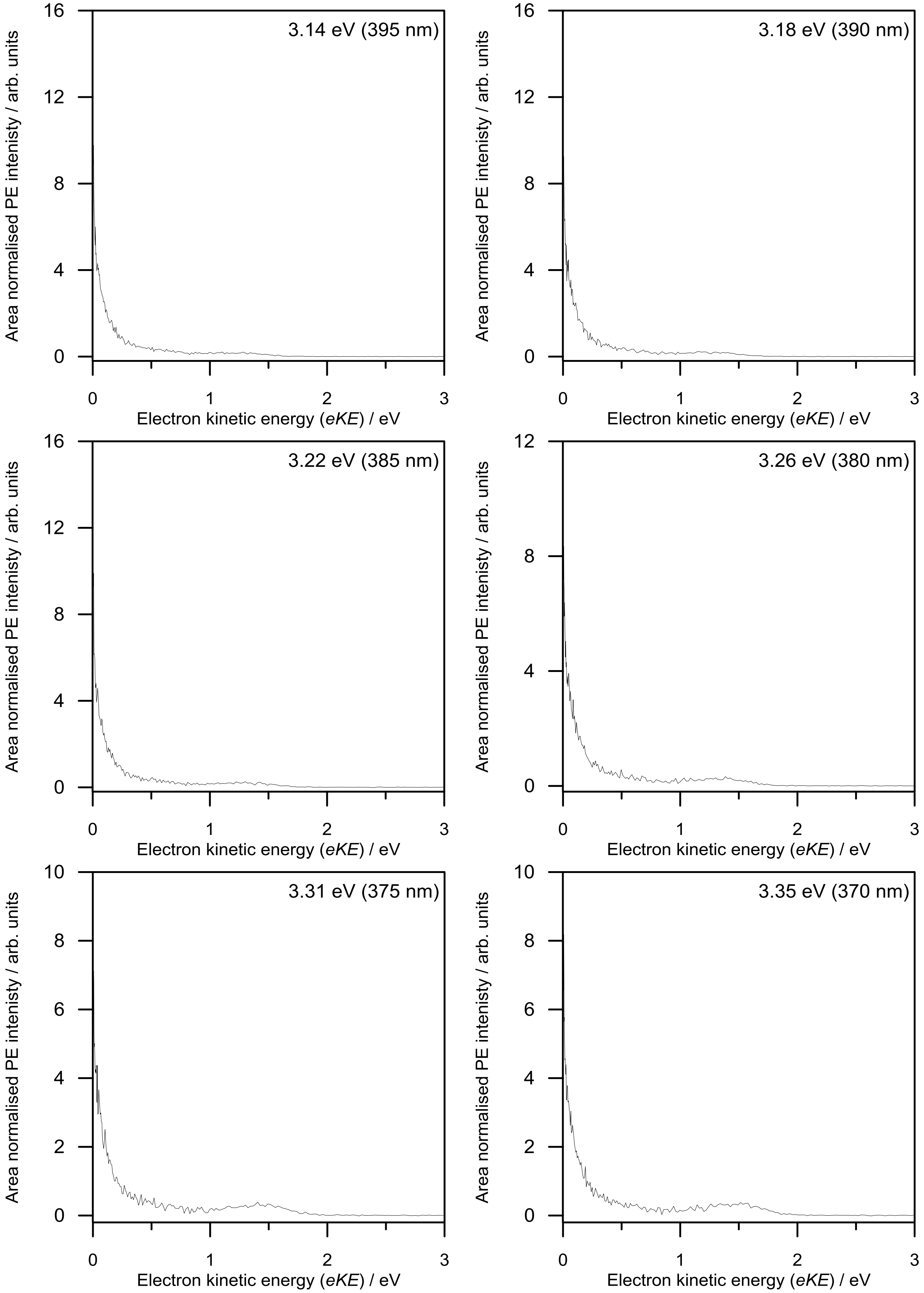
3.14 eV (395 nm)
3.18 eV (390 nm)
3.22 eV (385 nm)
3.26 eV (380 nm)
3.31 eV (375 nm)
3.35 eV (370 nm)
Area normalised PE intenisty / arb. units
Electron kinetic energy (eKE) / eV

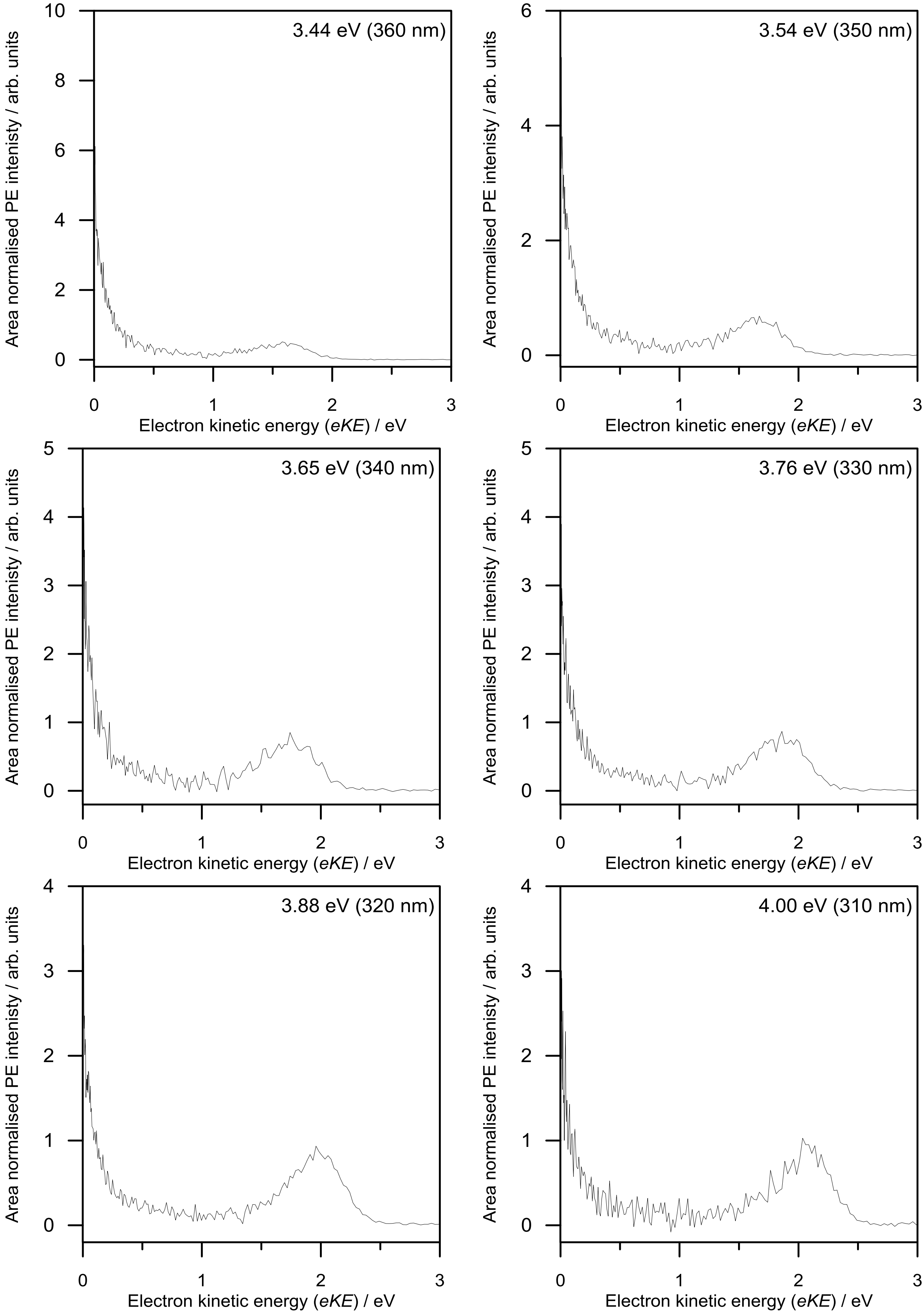

3.44 eV (360 nm)
3.54 eV (350 nm)
3.65 eV (340 nm)
3.76 eV (330 nm)
3.88 eV (320 nm)
4.00 eV (310 nm)
Area normalised PE intenisty / arb. units
Electron kinetic energy (eKE) / eV

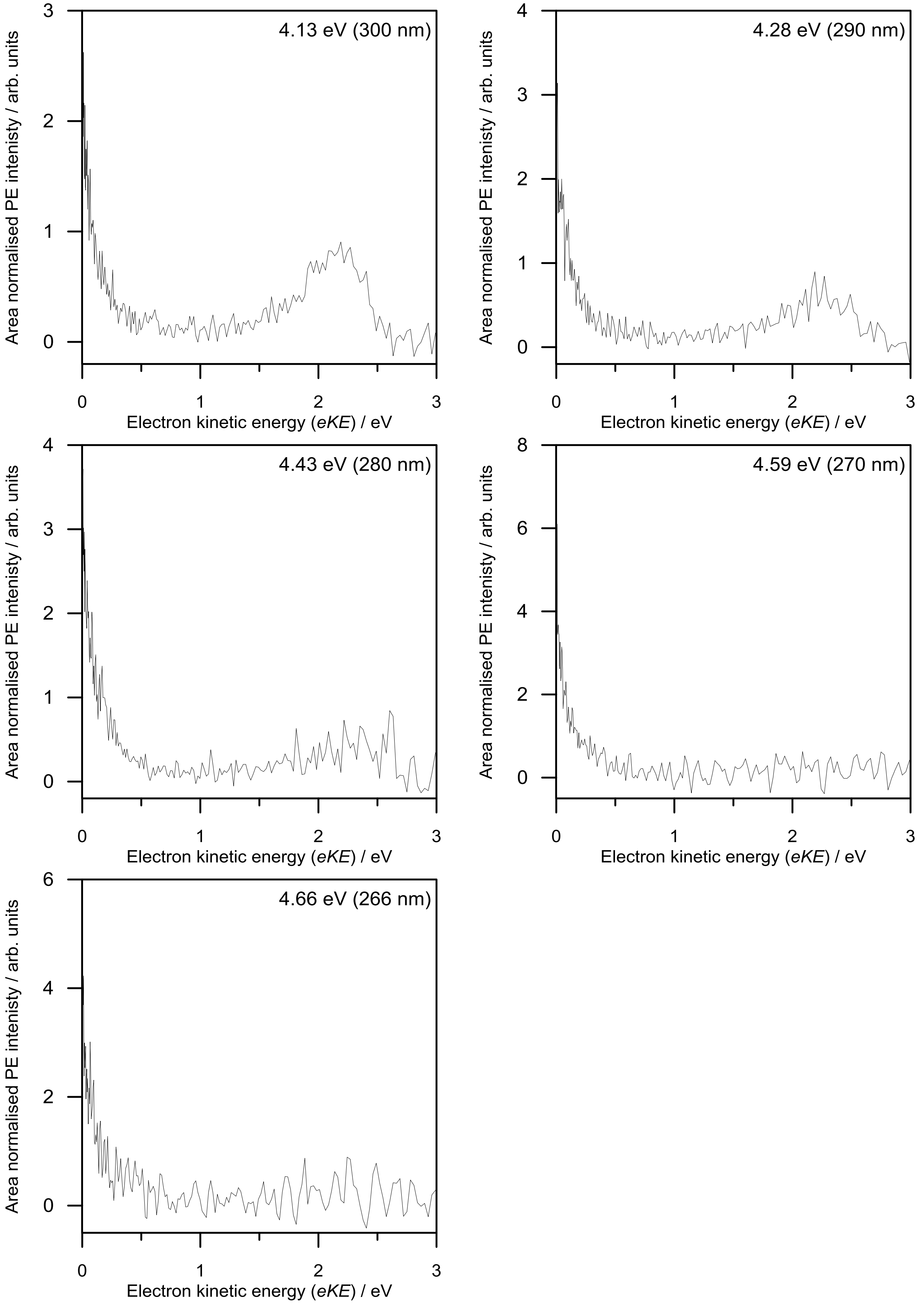
4.13 eV (300 nm)
4.28 eV (290 nm)
4.43 eV (280 nm)
4.59 eV (270 nm)
4.66 eV (266 nm)
Area normalised PE intenisty / arb. units
Electron kinetic energy (eKE) / eV

Example time-resolved photoelectron spectra for the 3.10 eV + 1.55 eV (400 nm + 800 nm) pump-probe scheme illustrating the A3 and A2* components

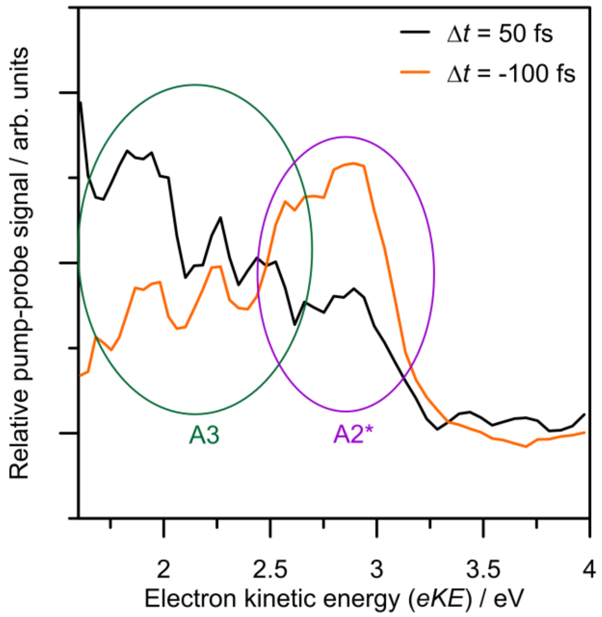

Supplement: Supplementary file 1 [file SC-006-C4SC03491K-s001.pdf]
